# Liver‐Targeted Gallium‐Polyphenol Network by Disrupting the ROS/NETs/PANoptosis Axis for Precision Acute Liver Injury Therapy

**DOI:** 10.1002/advs.202524135

**Published:** 2026-03-26

**Authors:** Xiaopeng Cai, Jian He, Jingwen Deng, Ziwei Wang, Zhongshu Wu, Kaiyue Wang, Xiang Zheng, Zhenzhen Gao, Xi Ma, Shumei Wei, Xiangrong Hu, Yuan Ding, Weilin Wang, Min Zhou

**Affiliations:** ^1^ Hepatobiliary and Pancreatic Surgery The Second Affiliated Hospital Zhejiang University School of Medicine Hangzhou Zhejiang China; ^2^ Zhejiang University‐University of Edinburgh Institute (ZJU‐UoE Institute) and Liangzhu Laboratory Zhejiang University School of Medicine Zhejiang University Haining Zhejiang China; ^3^ Zhejiang University‐Ordos City Etuoke Banner Joint Research Center Zhejiang University Haining Zhejiang China; ^4^ Department of Medical Oncology Sir Run Run Shaw Hospital School of Medicine Zhejiang University Hangzhou Zhejiang China; ^5^ Zhejiang University‐University of Edinburgh Institute (ZJU‐UoE Institute) Zhejiang University Haining China; ^6^ Department of Radiology Sir Run Run Shaw Hospital of School of Medicine Zhejiang University Hangzhou Zhejiang China; ^7^ Department of surgery and International Institutes of Medicine The Fourth Affiliated Hospital Zhejiang University School of Medicine Yiwu Zhejiang China; ^8^ Departments of Clinical Pathology The Second Affiliated Hospital of Medical College of Zhejiang University Hangzhou Zhejiang China

**Keywords:** acute liver injury, gallium, neutrophil extracellular traps, PANoptosis, polyphenol, reactive oxygen species

## Abstract

Acute liver injury (ALI), driven by diverse insults such as drug toxicity and ischemia‐reperfusion, poses a high mortality risk and lacks targeted therapies. While reactive oxygen species (ROS), neutrophil extracellular traps (NETs), and a coordinated cell death pathway PANoptosis have been implicated, their interplay as a unified pathogenic axis remains elusive. Here, by integrating multi‐omics analyses of clinical databases and patient samples, we systematically identified and validated the ROS/NETs/PANoptosis axis as a central driver of hepatocyte damage across multiple ALI etiologies. To therapeutically target this axis, we engineered a liver‐targeted gallium‐quercetin nanocomposite (Ga@Que) via coordination‐driven self‐assembly. Ga@Que effectively overcomes the poor bioavailability of natural quercetin. In murine models of acetaminophen‐induced and ischemia‐reperfusion liver injury, Ga@Que exhibited significant liver accumulation, potently scavenged ROS, suppressed neutrophil infiltration and NETs formation, and attenuated PANoptosis. Consequently, Ga@Que treatment markedly mitigated liver damage and inflammation, outperforming its individual components. Our study not only delineates a novel pathogenic paradigm in ALI but also introduces Ga@Que as a promising precision nanotherapeutic, offering a synergistic and translatable strategy to disrupt this deleterious cascade.

## Graphical abstract

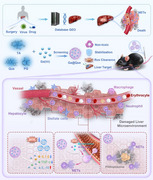

## 1 Introduction

Acute liver injury (ALI) is a life‐threatening clinical syndrome characterized by rapid hepatocyte necrosis, leading to high mortality and an urgent need for liver transplantation. Its etiology is diverse, encompassing drug overdose (e.g., acetaminophen), viral hepatitis, and surgical insults such as hepatic ischemia‐reperfusion injury (HIRI) [1, 2, 3]. The pathophysiology of ALI is complex, primarily driven by uncontrolled oxidative stress and a dysregulated immune‐inflammatory response [4, 5]. Although the antioxidant N‐acetylcysteine (NAC) is clinically used, its efficacy is limited by a narrow therapeutic window and lack of targeted delivery [6, 7]. Consequently, elucidating the precise mechanisms driving ALI progression and developing novel targeted therapeutics remain paramount challenges.

In recent years, neutrophil extracellular traps (NETs)—network‐like structures of DNA, histones, and granular proteins released by activated neutrophils—have emerged as a double‐edged sword in immunity. While crucial for pathogen clearance, excessive NETs formation can inflict collateral damage on host tissues, propagating inflammation and contributing to the pathogenesis of various diseases, including autoimmune diseases, tumor and chronic liver conditions [8, 9, 10, 11, 12, 13]. Growing evidence also implicates NETs in ALI [14, 15, 16]. However, a systematic evaluation of their role across different ALI etiologies and a clear understanding of their upstream triggers and downstream effectors are still lacking.

Reactive oxygen species (ROS) are pivotal upstream signaling molecules that potently induce NETosis (NETs formation) in numerous pathological contexts [17, 18, 19, 20]. In ALI, massive ROS burst from damaged hepatocytes and infiltrating immune cells is a hallmark event. While the interplay between ROS and NETs has been suggested in liver disease [21, 22, 23], their causal relationship and concerted action in ALI pathogenesis require further validation. Furthermore, the mechanistic link connecting NETs to ultimate hepatocyte death is poorly defined [24]. PANoptosis, a novel and unique inflammatory programmed cell death pathway integrating the key features of pyroptosis, apoptosis, and necroptosis, has recently been identified as a critical executor in liver diseases [25, 26, 27, 28, 29]. We hypothesized that a pathogenic axis centered on ROS, NETs, and PANoptosis may drive the vicious cycle of hepatocyte death and inflammation in ALI. Yet, the existence and therapeutic relevance of this ROS/NETs/PANoptosis axis remain largely unexplored.

Natural polyphenols like quercetin (Que) represent promising therapeutic candidates due to their potent antioxidant and anti‐inflammatory properties [30, 31, 32, 33]. However, their clinical translation is severely hampered by poor water solubility and low bioavailability. Metal‐phenolic networks (MPNs), formed by the coordination of metal ions with polyphenols, have recently emerged as a versatile nanoplatform to enhance the stability and bioactivity of polyphenols [34, 35, 36]. Notably, gallium (Ga) ions possess inherent immunomodulatory properties, and Ga‐based formulations have proven clinical safety profiles [37]. We reasoned that a Ga‐based MPN could synergize the advantages of both components.

**SCHEME 1:**
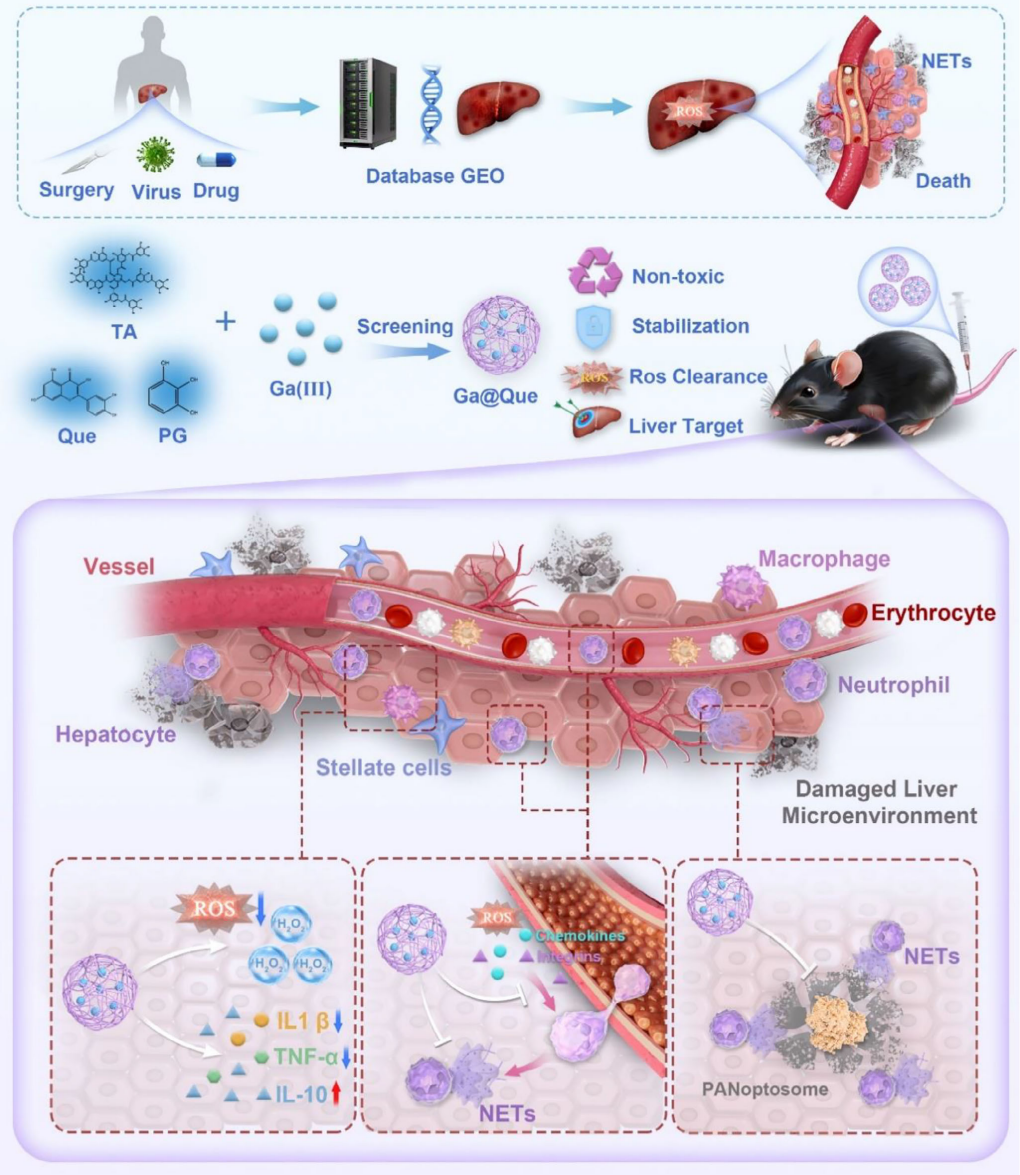
Schematic illustration of the Ga@Que nanocomposite alleviating acute liver injury (ALI) by disrupting the ROS/NETs/PANoptosis axis. The nanocomposite is formed by coordinating gallium ions (Ga^3^^+^) with the natural polyphenol quercetin (Que). Upon intravenous administration, Ga@Que preferentially accumulates in the injured liver. There, it scavenges reactive oxygen species (ROS), which in turn inhibits the formation of neutrophil extracellular traps (NETs) by infiltrating neutrophils. The suppression of NETs blocks the subsequent activation of PANoptosis—an integrated cell death pathway combining pyroptosis, apoptosis, and necroptosis—thereby protecting hepatocytes and mitigating liver damage.

Herein, we first systematically validated the ROS/NETs/PANoptosis axis as a common pathogenic driver in multiple forms of ALI through integrated analysis of multi‐omics databases, clinical samples, and murine models. To therapeutically target this axis, we then engineered a liver‐targeted gallium‐quercetin nanocomposite (Ga@Que). This nanotherapeutic was designed to simultaneously overcome the limitations of Que and leverage the synergistic effects of Ga and Que. We demonstrated that Ga@Que effectively accumulates in the liver, scavenges ROS, inhibits NETs formation, and blocks PANoptosis, ultimately conferring potent protection against ALI (Scheme 1). Our work not only unveils a critical pathogenic axis in ALI but also provides a precision nanomedicine strategy for its treatment.

## 2 Results

### 2.1 A Unified Axis in Human ALI: ROS, NETs, and PANoptosis

**FIGURE 1:**
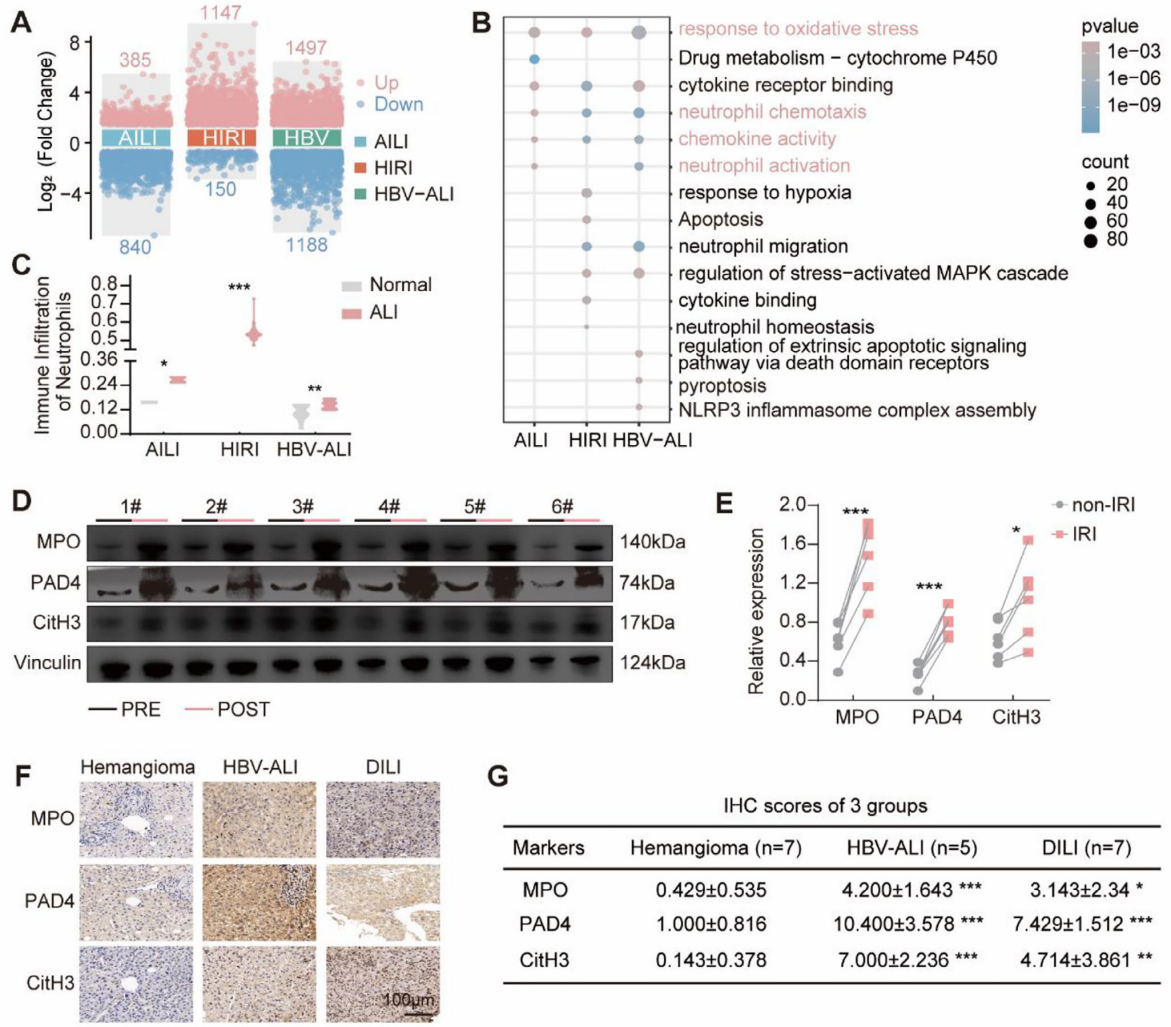
NETs are present in multiple forms of human acute liver injury (ALI). (A) Differentially expressed genes (DEGs) in datasets GSE120652 (AILI), GSE38941 (HBV‐ALI), GSE151648 (HIRI). (B) Gene Ontology (GO) and Kyoto Encyclopedia of Genes and Genomes (KEGG) analysis of DEGs across the three ALI types. (C) Immune infiltration of neutrophils assessed by single sample gene set enrichment analysis (ssGSEA) algorithm in the indicated datasets. (D, E) Western blot analysis (E) and quantification (F) of NETs markers (MPO, PAD4, CitH3) in paired liver samples from patients before and after portal clamping during surgery (n = 6). (F, G) Representative IHC staining (F) and IHC scores (G) of MPO, PAD4, and CitH3 in liver from control (patients with hemangioma, n = 7) compared to those with HBV‐ALI (n = 5) and DILI (n = 7). *p< 0.05, **p< 0.01, ***p< 0.001.

To investigate the universal mechanisms underlying ALI, we initiated our study with an integrated analysis of public transcriptomic datasets and clinical samples from patients with AILI, HIRI, and HBV‐ALI. This unbiased approach revealed a consistent signature of dysregulated oxidative stress, robust chemokine activity, and significant neutrophil infiltration across all three ALI types (Figure 1A‐C). This finding prompted us to focus on a key effector of neutrophils—NETs. Indeed, we detected a marked increase in NETs markers (MPO, PAD4, CitH3) in liver tissues from patients following portal clamping during surgery, as well as in samples from AILI and HBV‐ALI patients (Figure 1D‐G). These data firmly established the presence of excessive NETs formation as a common pathological feature in human ALI.

**FIGURE 2:**
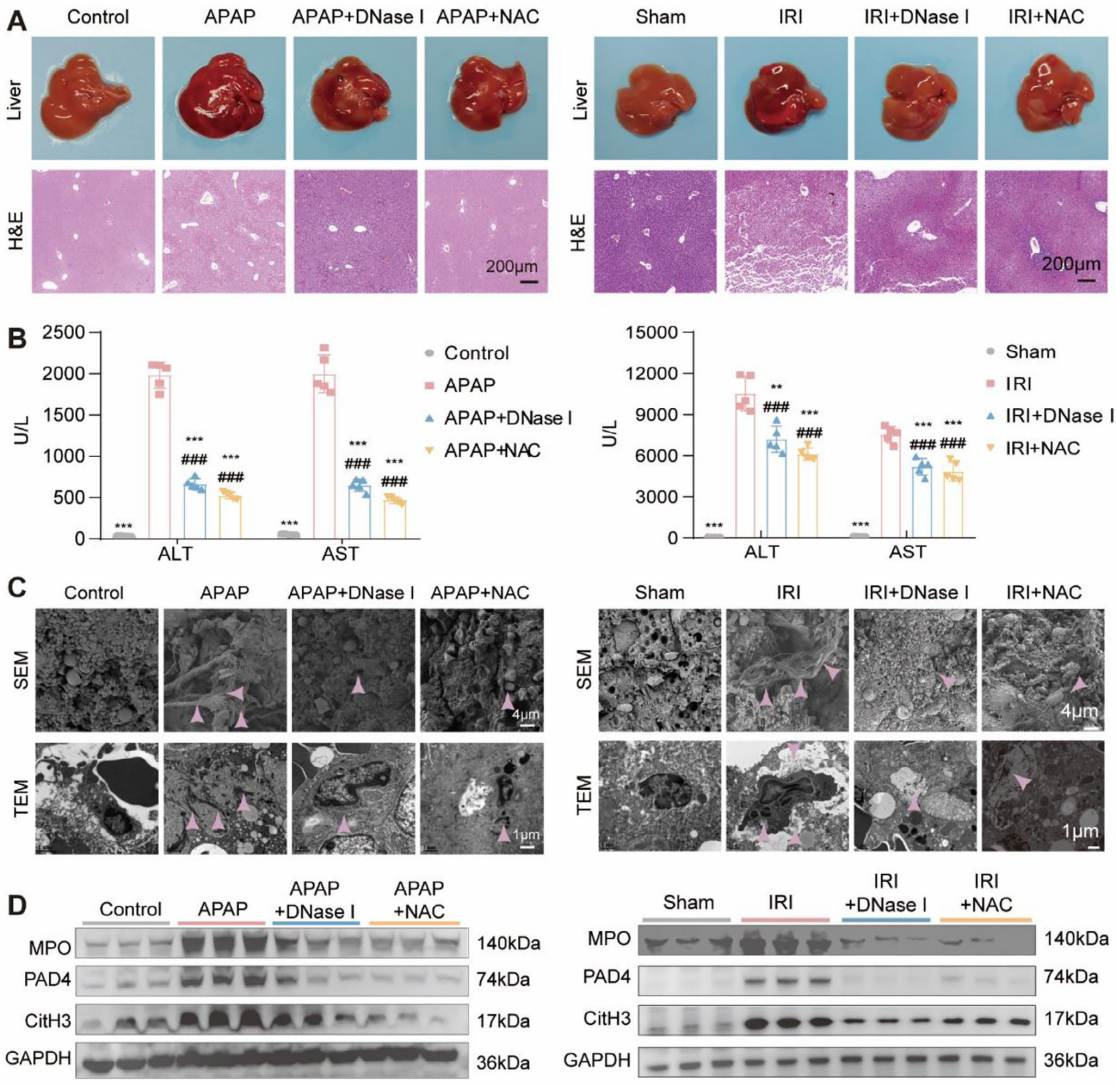
Blocking ROS‐mediated NETs formation protects against acute liver injury (ALI). (A) Liver macroscopic appearance and HE staining in ALI mice (n = 5). (B) Levels of alanine aminotransferase (ALT) and aspartate aminotransferase (AST) (n = 5). (C) Representative transmission electron microscopy (TEM) and scanning electron microscopy (SEM) images showing NETs structures (indicated by pink arrows) in liver tissues. (D) Western blot analysis of NETs markers (MPO, PAD4, CitH3) in liver tissues (n = 3). *p < 0.05, **p < 0.01, ***p < 0.001 vs. APAP (or IRI) group; #p < 0.05, ##p < 0.01, ###p < 0.001 vs. Control (or Sham) group.

Given the concurrent oxidative stress signature, we hypothesized that ROS might be a critical upstream trigger for NETs in ALI. To validate this, we employed well‐established AILI and HIRI mouse models. Scavenging ROS with NAC or dismantling NETs with DNase I significantly attenuated liver injury, as evidenced by improved histology and reduced serum alanine aminotransferase (ALT) and aspartate aminotransferase (AST) levels (Figure 2A,B). Crucially, both interventions potently suppressed the formation of NETs, as visualized by electron microscopy and confirmed by reduced expression of MPO, PAD4, and CitH3 (Figure 2C, D; Figure S1A, B). We conclude that ROS‐driven NETs formation is a pivotal pathogenic event in ALI of varying etiologies.

### 2.2 From NETs to Cell Death: Orchestrating PANoptosis

**FIGURE 3:**
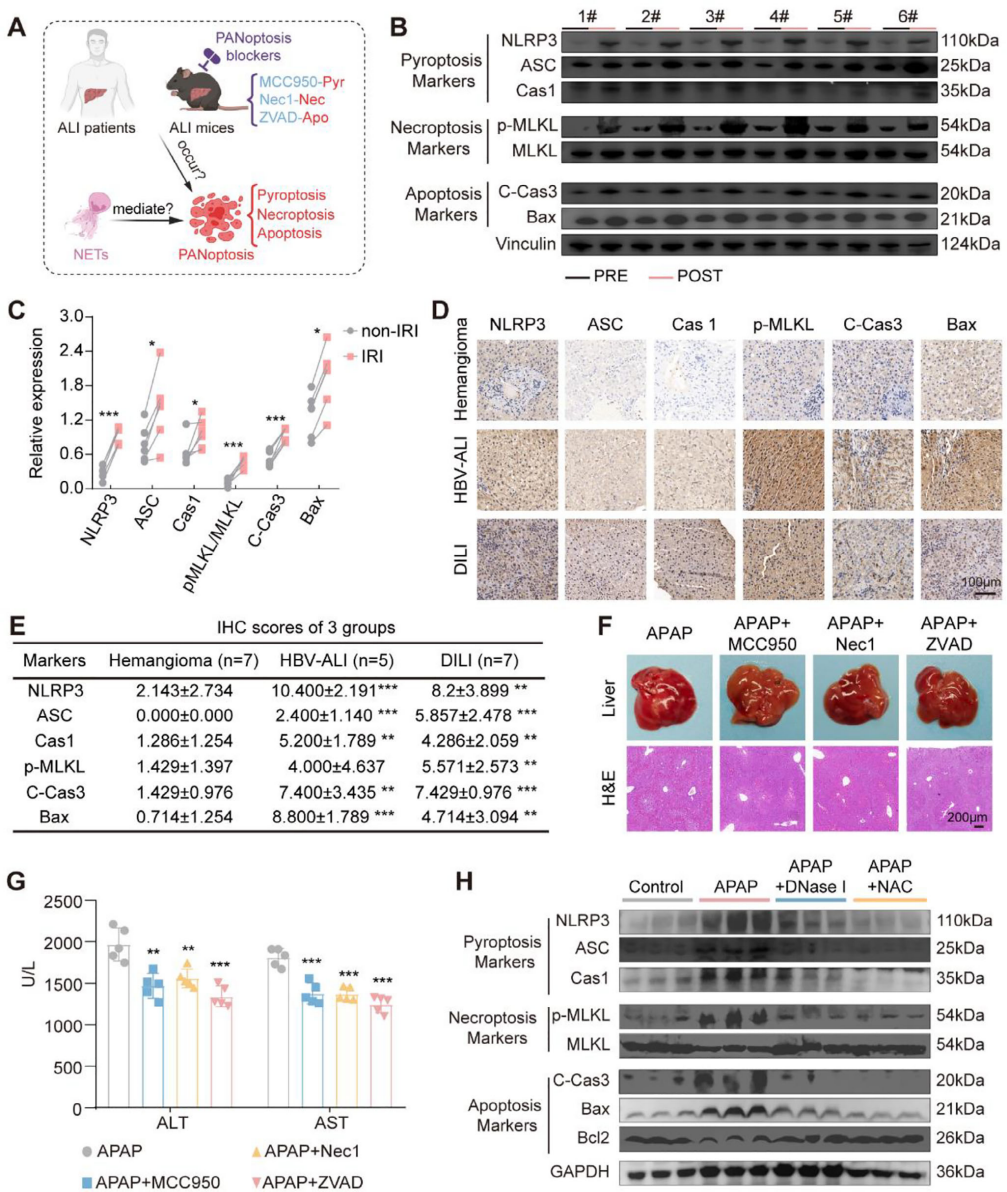
NETs mediate PANoptosis in acute liver injury (ALI). (A) Workflow for validating NETs‐mediated PANoptosis in clinical samples and mouse models. Created with BioRender (https://biorender.com). (B, C) Western blot analysis (B) and quantification (C) of PANoptosis markers in paired liver samples from hepatic ischemia‐reperfusion injury (HIRI) patients (n = 6). (D, E) Representative IHC staining (D) and IHC scores (E) of PANoptosis markers in liver sections from control (patients with hemangioma, n = 7) compared to those with HBV‐ALI (n = 5) and DILI (n = 7). (F) Liver macroscopic appearance and HE staining in acetaminophen‐induced liver injury (AILI) mice treated with PANoptosis inhibitors (n = 5). (G) Levels of alanine aminotransferase (ALT) and aspartate aminotransferase (AST) in mice from the indicated groups, compared with the APAP model group (n = 5). (H) Western blot analysis of PANoptosis markers in liver tissues from AILI mice treated with NAC or DNase I (n = 3). *p< 0.05, **p< 0.01, ***p< 0.001.

Having established ROS‐mediated NETs as a key driver, we sought to identify the primary downstream executor of hepatocyte damage. PANoptosis, an integrated inflammatory cell death pathway, emerged as a compelling candidate (Figure 3A). Analysis of clinical samples confirmed a significant upregulation of PANoptosis markers in HIRI, AILI, and HBV‐ALI patients (Figure 3B‐E). To functionally validate its role, we administered specific inhibitors of apoptosis (Z‐VAD‐FMK), necroptosis (Nec‐1), and pyroptosis (MCC950) in AILI and HIRI mice. All inhibitors conferred significant protection against liver injury (Figure 3F,G; Figure S2A, B), confirming the operational involvement of PANoptosis in ALI.

Most importantly, disrupting the upstream steps by scavenging ROS with NAC or degrading NETs with DNase I effectively suppressed the activation of all three PANoptosis branches in vivo (Figure 3H; Figure S2C‐E). To directly assess the impact of NETs on hepatocytes in vitro, exposing Hep G2 cells to isolated NETs significantly increased the expression of ASC, p‐MLKL, and Bax, while clearance of NETs downregulated the expression of PANoptosis markers (Figure S2F‐H). These results demonstrate that NETs act as a crucial upstream mediator that orchestrates PANoptosis in ALI, solidifying the ROS/NETs/PANoptosis axis.

### 2.3 An Engineered Solution: Liver‐Targeted Ga@Que Nanocomposites

**FIGURE 4:**
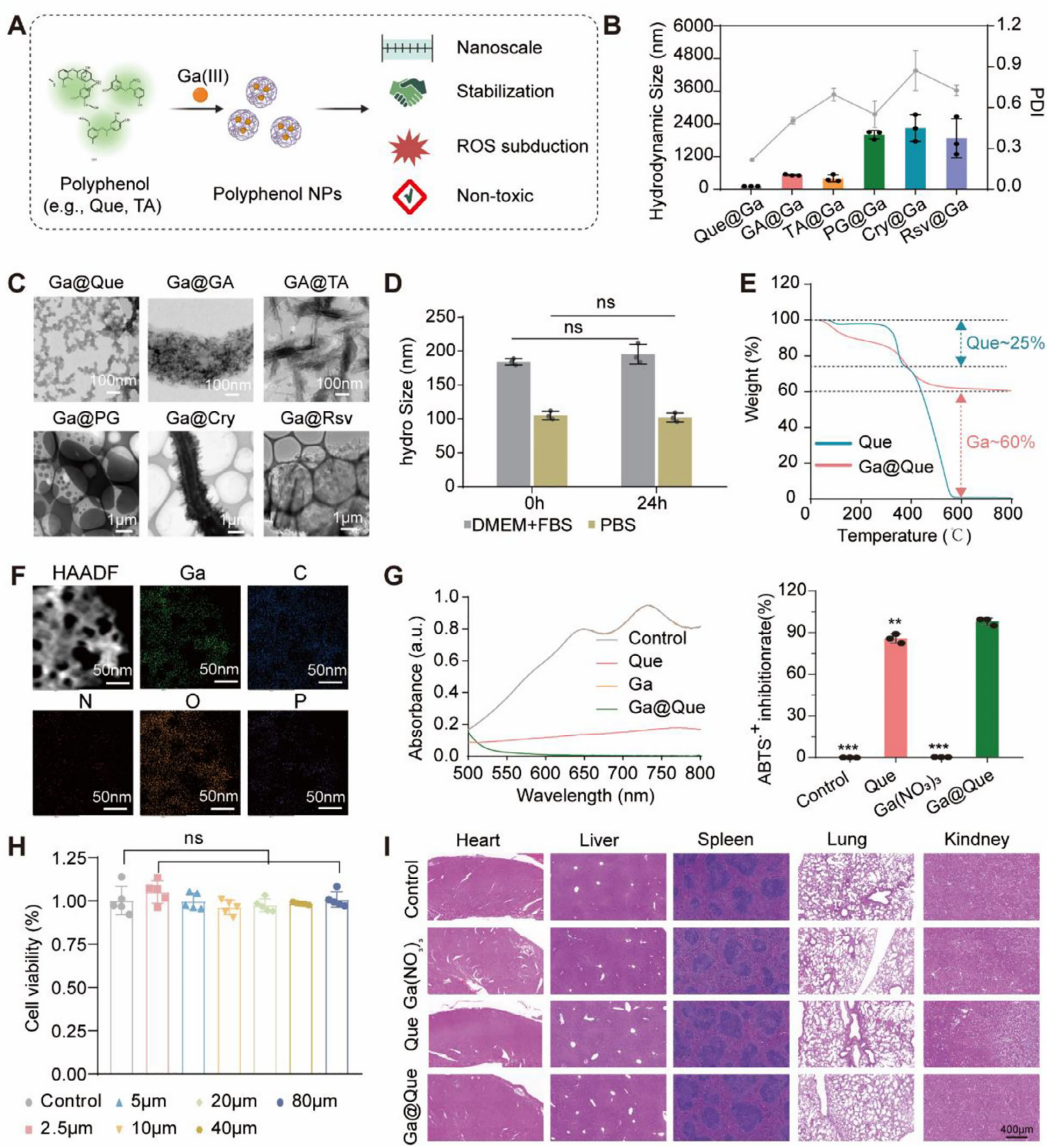
Synthesis, characteristics and biosafety of Ga@Que. (A) Schematic of the screening and synthesis process for gallium‐polyphenol nanocomposites. Created with BioRender (https://biorender.com). (B) Hydrodynamic size and polydispersity index (PDI) of Ga@polyphenol complexes (n = 3). (C) TEM images of Ga@polyphenol complexes (D) Hydrodynamic size change in culture medium (DMEM+FBS) and PBS (n = 3). (E) Thermogravimetric analysis of Que and Ga@Que. (F) Elemental distribution in Ga@Que. (G) ABTS^+^ scavenging assay absorbance curves and inhibition rates, compared with the Ga@Que group (n = 3). (H) Cell viability of Hep G2 cells treated with Ga@Que for 24 h (n = 5). (I) Organ pathology for short‐term (24 h) biosafety evaluation of Ga(NO_3_)_3_, Que, Ga@Que. **p< 0.01, ***p< 0.001.

The critical role of the ROS/NETs/PANoptosis axis highlighted it as a promising therapeutic target. To overcome the limitations of conventional antioxidants like NAC, we engineered a targeted nanotherapeutic. We screened a library of gallium‐polyphenol coordination complexes, including Quercetin (Que), Gallic Acid (GA), Tannic Acid (TA), Pyrogallol (PG), Chrysin (Cry), Resveratrol (Rsv) (Figure 4A). Among them, the gallium‐quercetin nanocomposite (Ga@Que) displayed optimal characteristics: the smallest hydrodynamic diameter (∼120 nm), lowest polydispersity (PDI ∼0.21), and excellent colloidal stability in physiological solutions (Figure 4B‐D; Figure S3A, B). Comprehensive characterization (TEM, EDS, XPS, FTIR, TGA) confirmed the successful formation of uniform, spherical nanoparticles with strong Ga‐Que coordination and the desired composition (Figure 4E,F; Figure S3C‐F).

Leveraging this enhanced stability, Ga@Que exhibited superior free radical scavenging capacity against multiple ROS species compared to free Que (Figure 4G; Figure S4A‐C). It demonstrated excellent biosafety, showing no significant cytotoxicity in vitro (Figure 4H) and no adverse effects on major organs or serum biochemistry in vivo over both short and long terms (Figure 4I; Figure S4D‐F). Furtherly, in vitro release of Ga^3^^+^ in PBS remained below 3% over 7 days as measured by ICP‑MS, confirming the high stability of the coordination network (Figure S4G). In vivo, gallium levels in the liver of healthy mice decreased by less than 2% over 7 days post‑injection, with no prolonged accumulation in non‑target organs and gradual systemic clearance (Figure S4H). Thus, Ga@Que was identified as a stable, safe, and highly effective ROS‐scavenging nanoplatform ideal for further investigation.

### 2.4 Targeted Delivery and Efficacy: Ga@Que Confers Potent Protection

**FIGURE 5:**
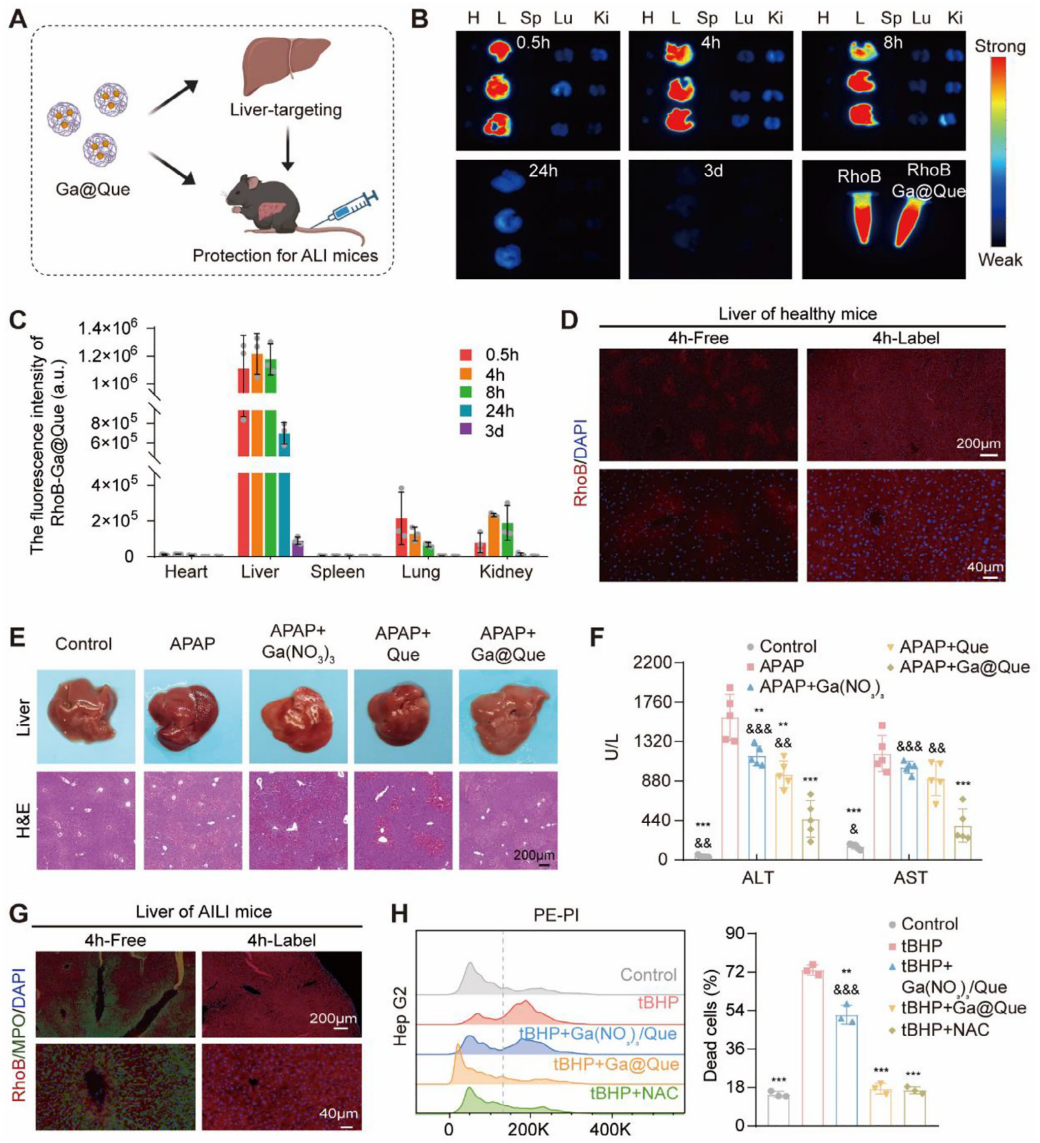
Liver‐targeted accumulation and protective efficacy of Ga@Que in acute liver injury (ALI). (A) Schematic of the experimental design for evaluating biodistribution and therapeutic effect. Created with BioRender (https://biorender.com). (B, C) Ex vivo fluorescence imaging (B) and semi‐quantification (C) of RhoB signals in major organs (H, heart; L, liver; S, spleen; Lu, lung; K, kidney) at indicated time points after RhoB‐Ga@Que injection (n = 3). (D) Immunofluorescence images of liver sections from healthy mice injected with free RhoB or RhoB‐Ga@Que. Nuclei were stained with DAPI (blue). (E) Liver macroscopic appearance and HE staining in acetaminophen‐induced liver injury (AILI) mice (n = 5). (F) Alanine aminotransferase (ALT) and aspartate aminotransferase (AST) levels in AILI mice (n = 5). (G) Immunofluorescence images of liver sections from AILI mice, showing RhoB‐Ga@Que (red) localization and MPO (green) expression. (H) Live/Dead evaluation of Hep G2 cells subjected to tBHP‐induced injury and treated with the indicated agents (n = 3). **p < 0.01, ***p < 0.001 vs. APAP (or tBHP) group; &p < 0.05, &&p < 0.01, &&&p < 0.001 vs. APAP (or tBHP) + Ga@Que group.

We next evaluated the biodistribution and therapeutic efficacy of Ga@Que (Figure 5A). Following intravenous administration, Rhodamine B‐labeled Ga@Que (RhoB‐Ga@Que) showed rapid and predominant accumulation in the liver (L), with signals persisting significantly longer than in other organs, such as heart (H), spleen (Sp), lungs (Lu), and kidneys (Ki) (Figure 5B‐D). Over 24 h to three days, fluorescence signals gradually diminished, but residual signal persisted in the liver, suggesting sustained retention. This inherent liver‐targeting property is ideal for treating ALI.

In both AILI and HIRI mouse models, Ga@Que treatment dramatically ameliorated liver injury, as indicated by macroscopic appearance, histology, and serum ALT/AST levels, outperforming its individual components (Ga(NO_3_)_3_ or Que alone) (Figure 5E,F; Figure S5A, B). Immunofluorescence confirmed the co‐localization of Ga@Que with sites of oxidative stress (MPO) in injured livers (Figure 5G). In a cellular model of oxidative injury, Ga@Que effectively protected Hep G2 cells from tBHP‐induced death, similar to the positive control NAC (Figure 5H).

While NAC remains the standard treatment for APAP overdose, its therapeutic efficacy diminishes when administered beyond the early treatment window (>3 h post‑overdose) [6]. In contrast, Ga@Que continued to demonstrate significant hepatoprotective effects even under such delayed‑treatment conditions and outperformed NAC in AILI mice (Figure S5C, D). These findings underscore the extended therapeutic potential of Ga@Que beyond the conventional treatment window.

### 2.5 Ga@Que Suppresses Oxidative Stress and Inflammation

**FIGURE 6:**
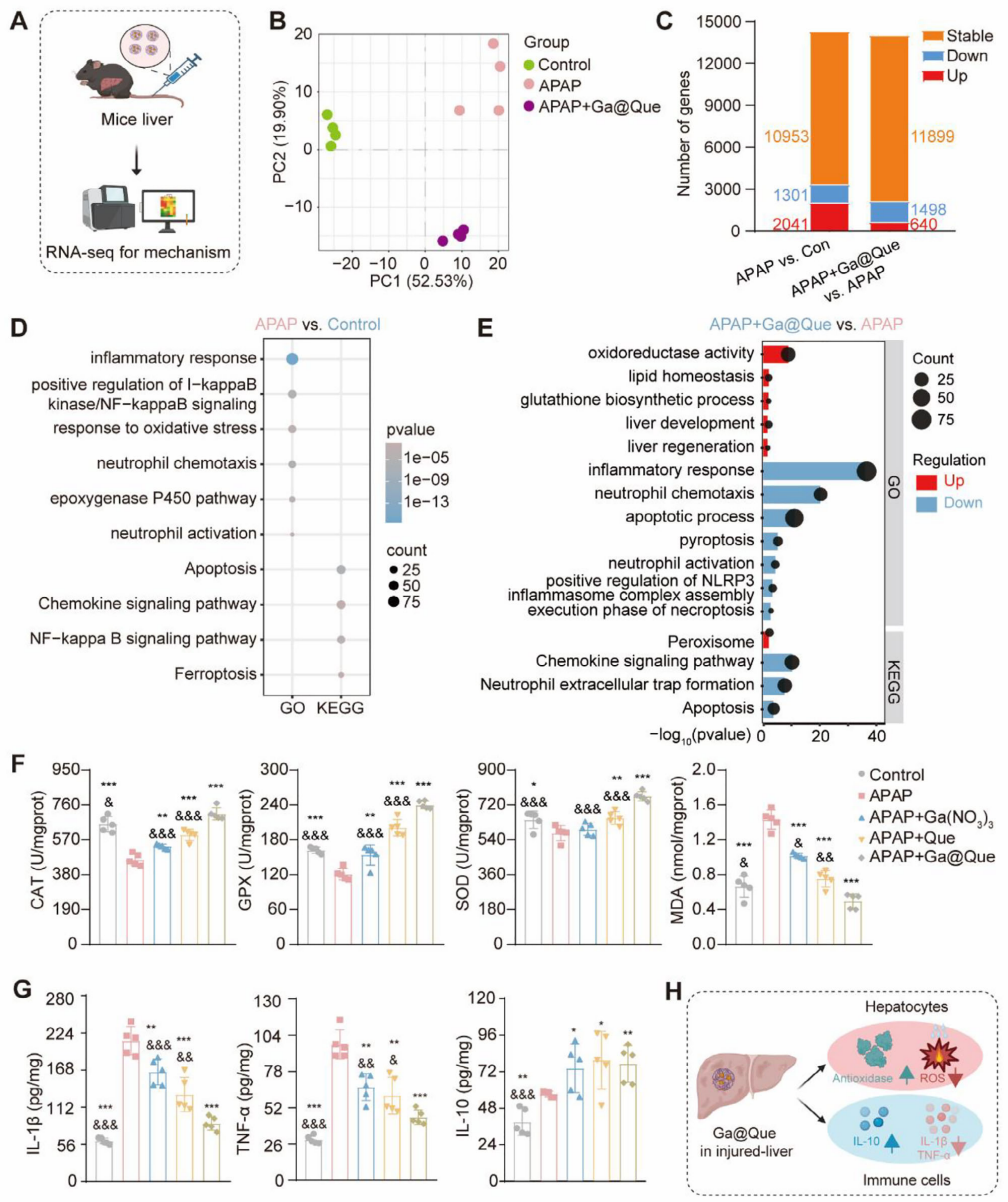
Ga@Que mitigates oxidative stress and inflammation in acute liver injury (ALI). (A) Workflow of the RNA sequencing (RNA‐seq) analysis in acetaminophen‐induced liver injury (AILI) mice. Created with BioRender (https://biorender.com). (B) PCA plot (n = 4). (C) Differentially expressed genes (DEGs) between different groups. (D, E) GO and KEGG pathway enrichment analysis of DEGs between APAP vs. Control (D) and APAP+Ga@Que vs. APAP (E) groups. (F) Oxidase levels (CAT, GPX, SOD) and MDA levels in AILI mice (n = 5). (G) Concentrations of inflammatory cytokines (IL‐1*β*, TNF‐*α*, IL‐10) in liver homogenates (n = 5). (H) Schematic summary of Ga@Que's effects on oxidative stress and inflammation. Created with BioRender (https://biorender.com). *p < 0.05, **p < 0.01, ***p < 0.001 vs. APAP group; &p < 0.05, &&p < 0.01, &&&p < 0.001 vs. APAP + Ga@Que group.

To unravel the mechanism of Ga@Que's protection, we performed RNA‐seq on liver tissues from AILI mice (Figure 6A). Transcriptomic profiling revealed distinct clustering of the Control, APAP, and APAP+Ga@Que groups (Figure 6B). APAP challenge triggered profound upregulation of genes involved in inflammatory responses, neutrophil chemotaxis, and cell death pathways (Figure 6C,D). Strikingly, Ga@Que treatment reversed these changes, significantly downregulating pathways related to oxidative stress and inflammation (Figure 6E).

Biochemical assays validated these findings: Ga@Que restored the activities of antioxidant enzymes (CAT, GPX, SOD), reduced lipid peroxidation (MDA), and decreased cellular ROS levels (Figure 6F; Figure S6A). Furthermore, it shifted the inflammatory milieu by lowering pro‐inflammatory cytokines (IL‐1*β*, TNF‐*α*) and elevating the anti‐inflammatory IL‐10 (Figure 6G; Figure S6B). Therefore, Ga@Que exerts its therapeutic effect by comprehensively mitigating oxidative stress and inflammation in the injured liver (Figure 6H).

### 2.6 Ga@Que Inhibits Neutrophil Recruitment and NETosis

**FIGURE 7:**
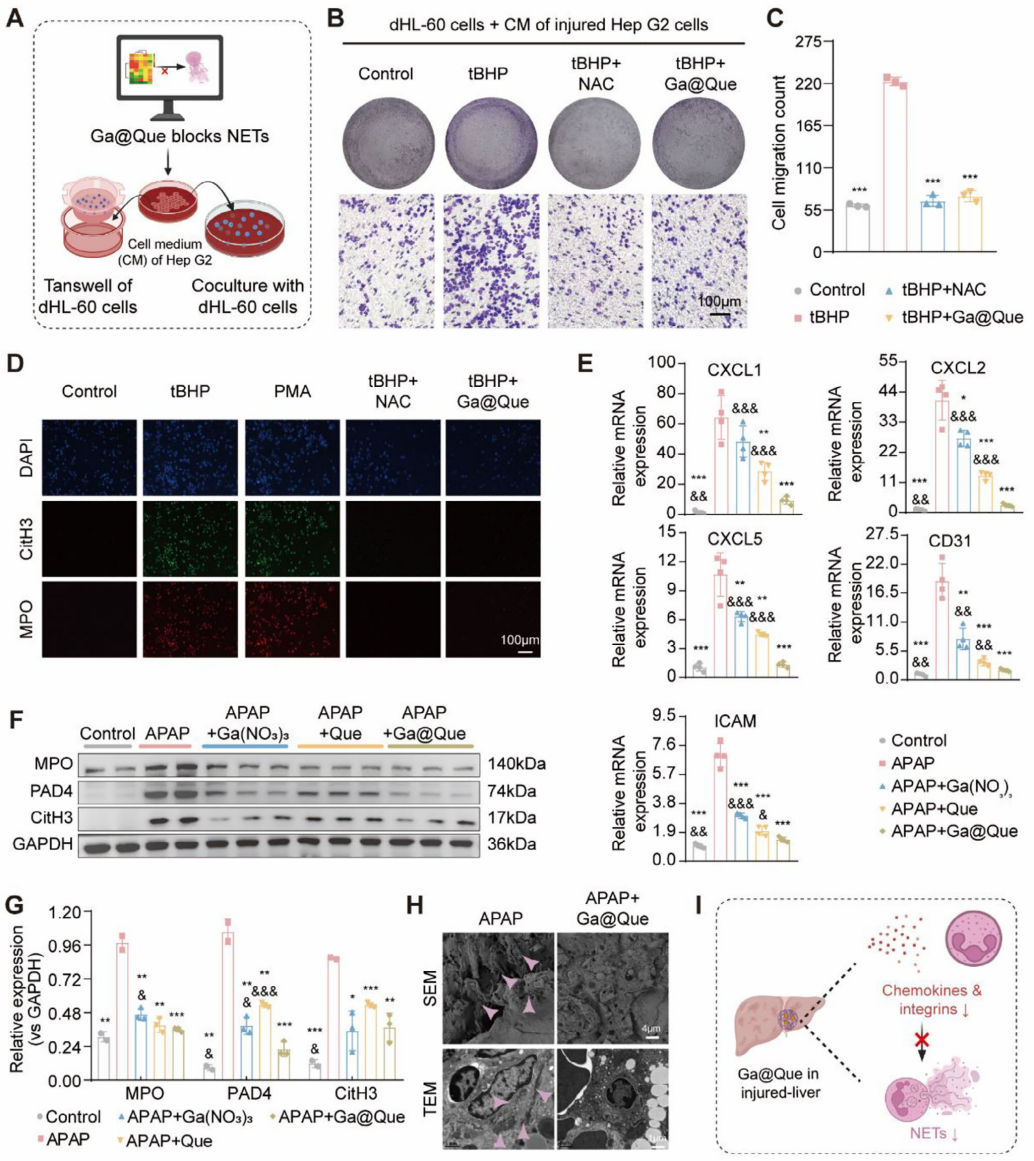
Ga@Que blocks neutrophil migration and NETs formation in acute liver injury (ALI). (A) Schematic of the in vitro co‐culture and transwell migration assay. Created with BioRender (https://biorender.com). (B) Representative images of crystal violet‐stained dHL‐60 cells that migrated toward conditioned medium from injured Hep G2 cells. (C) Count of migratory dHL‐60 cells, compared with the tBHP group (n = 3). (D) Immunofluorescence images of dHL‐60 cells showing NETs formation (CitH3, green; MPO, red; DAPI, blue). (E) Relative mRNA expression of neutrophil chemokines and adhesion molecules in liver tissues of acetaminophen‐induced liver injury (AILI) mice (n = 4). (F, G) Western blot analysis (F) and quantification (G) of NETs markers in AILI mouse livers (n = 3). (H) Representative TEM and SEM images of NETs in liver tissues. (I) Schematic summary of Ga@Que's role in blocking neutrophil migration and NETosis. Created with BioRender (https://biorender.com). *p < 0.05, **p < 0.01, ***p < 0.001 vs. APAP (or tBHP) group; &p < 0.05, &&p < 0.01, &&&p < 0.001 vs. APAP (or tBHP) + Ga@Que group.

Our transcriptomic data indicated that Ga@Que suppressed neutrophil‐related pathways. We therefore investigated its impact on neutrophil migration and NETs formation, key steps in our proposed axis. In vitro, conditioned medium from tBHP‐injured Hep G2 cells potently attracted dHL‐60 cells and induced NETs formation; both effects were markedly inhibited by Ga@Que (Figure 7A‐D). In vivo, Ga@Que treatment significantly downregulated the expression of key neutrophil chemokines (CXCL1, CXCL2, CXCL5) and adhesion molecules (CD31, ICAM) (Figure 7E; Figure S7A). Consequently, Ga@Que dramatically reduced the protein levels of NETs markers (MPO, PAD4, CitH3) and the appearance of NETs structures as seen by electron microscopy in both ALI models (Figure 7F‐H; Figure S7B‐E). These results demonstrate that Ga@Que effectively breaks the pathogenic axis by limiting neutrophil influx and subsequent NETs formation (Figure 7I).

### 2.7 Ga@Que Attenuates NETs‐Mediated PANoptosis

**FIGURE 8:**
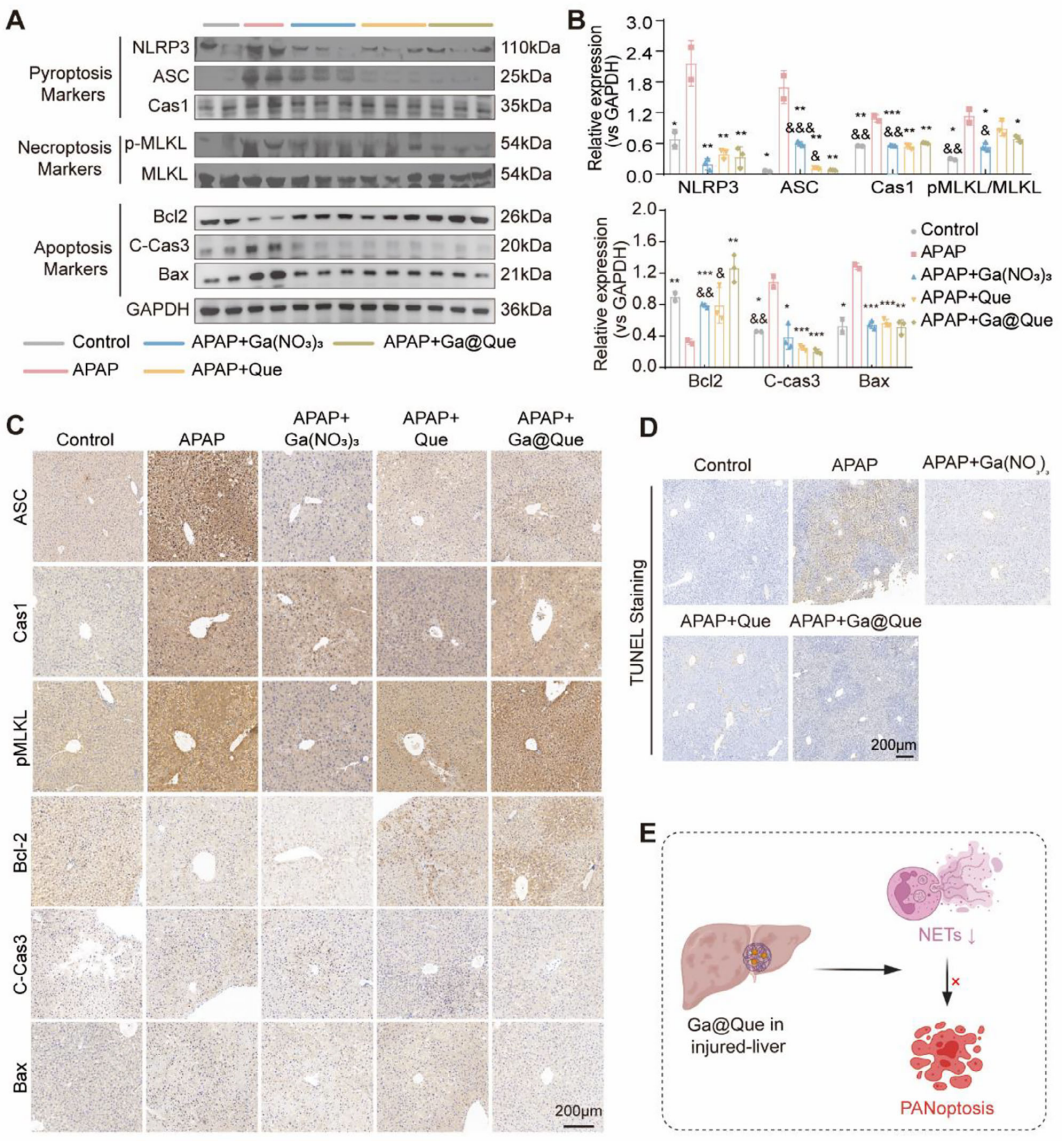
Ga@Que attenuates NETs‐mediated PANoptosis in acute liver injury (ALI). (A, B) Western blot analysis (A) and quantitative analysis (B) of PANoptosis markers in liver tissues from acetaminophen‐induced liver injury (AILI) mice (n = 3). (C) Immunohistochemical staining of PANoptosis biomarkers in AILI mice. (D) TUNEL staining in AILI mice. (E) Schematic diagram summarizing how Ga@Que reduces NETs‐mediated PANoptosis to protect against ALI. Created with BioRender (https://biorender.com). *p < 0.05, **p < 0.01, ***p < 0.001 vs. APAP group; &p < 0.05, &&p < 0.01, &&&p < 0.001 vs. APAP + Ga@Que group.

Finally, we examined the ultimate downstream event: PANoptosis. Ga@Que significantly reduced apoptosis in tBHP‐injured Hep G2 cells (Figure S8A, B). In AILI and HIRI mice, Ga@Que treatment led to a profound downregulation of key markers of apoptosis (Cleaved Caspase‐3, Bax/Bcl‐2), necroptosis (p‐MLKL), and pyroptosis (NLRP3, ASC, Caspase‐1) (Figure 8A‐C; Figure S8A‐C). This was further confirmed by a marked reduction in TUNEL‐positive cells (Figure 8D). Together, these data conclusively show that Ga@Que, by targeting the upstream ROS/NETs cascade, effectively blocks the execution of NETs‐mediated PANoptosis, thereby preserving hepatocyte viability and alleviating ALI (Figure 8E).

## 3 Discussion

Our study provides a paradigm shift in understanding and treating ALI by delineating a unified pathogenic axis and developing a precision nanotherapeutic to disrupt it. We first systematically identified and validated the ROS/NETs/PANoptosis axis as a common driver in diverse forms of human and murine ALI. Capitalizing on this mechanistic insight, we engineered Ga@Que, a liver‐targeted nano‐therapeutic that synergizes the anti‐inflammatory properties of gallium with the potent antioxidant capacity of quercetin. Ga@Que demonstrated superior efficacy in mitigating liver injury by concurrently scavenging ROS, inhibiting neutrophil recruitment and NETosis, and ultimately abrogating PANoptosis.

The role of NETs in chronic liver diseases is increasingly recognized [8, 9], but their systematic investigation across ALI etiologies has been limited [38, 39, 40]. Our integrated approach—spanning multi‐omics databases, clinical samples, and animal models—provides compelling evidence that NETs are not merely bystanders but active perpetrators in ALI pathophysiology. More importantly, we positioned NETs within a precise pathological cascade: they are triggered by the initial ROS burst and, in turn, act as the key upstream signal that orchestrates PANoptosis. This finding resolves a critical knowledge gap by linking innate immune activation (NETosis) to a coordinated cell death program in hepatocytes. The efficacy of DNase I and PANoptosis inhibitors in our models underscores the therapeutic relevance of this newly defined axis [41, 42, 43].

The limitations of current antioxidants like NAC necessitate innovative strategies [6]. While natural polyphenols like Que are promising, their poor bioavailability is a major translational hurdle [30, 31, 44, 45]. Our design of Ga@Que effectively overcomes this. The coordination‐driven self‐assembly into a nanoscale MPN was crucial, not only enhancing Que's stability and ROS‐scavenging capacity but also conferring passive liver‐targeting capabilities due to its ideal hydrodynamic size (∼120 nm), leveraging the sinusoidal fenestrations in the liver [46]. The choice of gallium was equally strategic, as its documented immunomodulatory properties likely contributed to the observed anti‐inflammatory effects, creating a synergistic “all‐in‐one” therapeutic platform [47, 48]. The robust liver accumulation and sustained retention of Ga@Que, as evidenced by in vivo imaging, directly translate to its potent protective effects.

Our RNA‐seq analysis offered an unbiased dissection of Ga@Que's mechanism. The transcriptomic landscape confirmed that Ga@Que reversal of APAP‐induced gene expression changes was predominantly associated with oxidative stress, neutrophil activation, and inflammatory cell death pathways. Subsequent biochemical and cellular assays validated that Ga@Que operates through a multi‐pronged mechanism: it directly quenches ROS, thereby resetting the hepatic redox balance, and indirectly dampens the inflammatory cascade by reducing pro‐inflammatory cytokines. This primary antioxidant and anti‐inflammatory effect created a hostile microenvironment for neutrophil recruitment, as shown by the downregulation of key chemokines and integrins, and the inhibition of dHL‐60 migration in vitro. The consequent reduction in NETs formation effectively broke the vicious cycle of the pathogenic axis.

Finally, we demonstrated that disrupting the upstream ROS/NETs cascade with Ga@Que effectively blocks the terminal executor of hepatocyte damage: PANoptosis. The comprehensive downregulation of markers for apoptosis, necroptosis, and pyroptosis confirms that Ga@Que intervention is capable of halting this integrated cell death process. This is significant because PANoptosis, by its very nature, is resistant to inhibitors targeting any single death pathway [28, 49, 50]. Our strategy of targeting a common upstream trigger (NETs) provides a more powerful and holistic approach to preserving hepatocyte mass [51, 52, 53].

Recent advances in ALI therapeutics, as summarized in Table S1, have introduced diverse strategies including natural polyphenol‐based modulation [54], redox‐responsive nanoparticle delivery [55], macrophage‐mediated approaches [56, 57], and liver‐specific CRISPR‐Cas editing [58]. These studies have advanced oxidative stress control, inflammation resolution, and targeted intervention in liver disorders.

Our Ga@Que nanocomposite complements these efforts by systematically validating the ROS/NETs/PANoptosis axis as a shared driver across ALI etiologies through multi‐omics and clinical analyses. The gallium‐quercetin coordination network markedly enhances polyphenol bioavailability and stability, while enabling synergistic ROS scavenging and immunomodulation. This results in robust hepatoprotection, excellent liver accumulation, and superior efficacy over individual components in both AILI and HIRI models.

Key limitations remain. The study relies on murine models; translation to larger animals and humans requires further pharmacokinetic, long‐term safety, and efficacy evaluation. Short‐term biosafety is confirmed, but long‐term effects, repeated dosing immunogenicity, and chronic‐use profiles are unaddressed. Laboratory‐scale synthesis may encounter challenges in GMP‐compliant large‐scale production and cost‐effectiveness. Additionally, detailed intracellular trafficking and drug interaction profiles warrant future investigation. These aspects highlight important next steps toward clinical development.

Overall, Ga@Que offers a mechanistically integrated, non‐cellular nanotherapeutic platform that extends the current landscape of precision ALI therapy.

## 4 Conclusions

In summary, our work unveils the ROS/NETs/PANoptosis axis as a unifying pathogenic mechanism in ALI and establishes Ga@Que as a potent, targeted nano‐therapeutic capable of disrupting this axis. By synergistically integrating the functionalities of gallium and quercetin into a single nanoplatform, Ga@Que overcomes the limitations of conventional treatments and natural products, offering a multi‐targeted and liver‐specific strategy. Our findings bridge a fundamental mechanistic insight with a transformative therapeutic innovation, paving the way for a new class of precision medicines for acute liver injury.

## 5 Materials and Methods

### 5.1 Public Data Acquisition and Analysis

Gene expression profiles from patients with acetaminophen‐induced liver injury (AILI, GSE120652), hepatic ischemia‐reperfusion injury (HIRI, GSE151648), and HBV‐associated ALI (HBV‐ALI, GSE38941) were retrieved from the Gene Expression Omnibus (GEO) database. Differential expression analysis was performed using the appropriate packages/platforms specific to each original dataset, with a significance threshold set at |log2 fold change| ≥ 1 and an adjusted p‐value < 0.05. Gene Ontology (GO) and Kyoto Encyclopedia of Genes and Genomes (KEGG) pathway enrichment analyses for the identified differentially expressed genes (DEGs) were conducted using the clusterProfiler R package (v4.0) [59]. Terms with an adjusted p‐value < 0.05 were considered significantly enriched. The relative abundance of 22 immune cell types in the liver tissue was estimated using the CIBERSORT algorithm [60].

### 5.2 Clinical Sample Collection

This study was conducted in accordance with the Declaration of Helsinki and the Declaration of Istanbul. The protocol was approved by the Ethics Committees of the Second Affiliated Hospital (Approval No. 2024‐1339) and Sir Run Run Shaw Hospital (Approval No. 2025‐1092) of Zhejiang University School of Medicine. Written informed consent was obtained from all participants. HIRI samples: Paired liver tissue samples (pre‐ and post‐ischemia) were collected from patients (n = 6) undergoing partial hepatectomy involving portal clamping. DILI samples: Archived paraffin‐embedded liver tissue samples from patients with drug‐induced liver injury (DILI, n = 5) were obtained from the Second Affiliated Hospital. HBV‐ALI and Control samples: Paraffin‐embedded liver tissues from patients with HBV‐ALI (n = 7) and control tissues from patients with hepatic hemangioma (n = 7) were acquired from Sir Run Run Shaw Hospital.

### 5.3 Animal Experiments

All animal protocols were approved by the Animal Research Committee of the Second Affiliated Hospital of Zhejiang University School of Medicine (Approval No. 2024‐234). Male C57BL/6J mice were obtained from Jiangsu Gempharmatech Co., Ltd. and maintained under specific pathogen‐free conditions. Both the AILI and HIRI mouse models were used. For AILI, mice received an intraperitoneal injection of 500 mg/kg acetaminophen (APAP) as described previously [61]. For HIRI, a 70% partial hepatic warm ischemia‐reperfusion injury (IRI) was induced [62]. In treatment group, DNase I (iv, 100 µg/mouse), NAC (ip, 100 mg/kg), Z‐VAD‐FMK (iv, 3 mg/kg), MCC950 (iv, 50 mg/kg), or Nec‐1 (iv, 3 mg/kg) were administered 30 min before model induction, with PBS served as the control. Additionally, mice were treated with Ga@Que (iv, 1 mg/kg) 30 min prior to model induction. Ga(NO_3_)_3_ and Que were given at a dose equivalent to that of Ga@Que. To investigate the post‐injury treatment windows and compare therapeutic efficacy with NAC, Ga@Que was administered 3 h after inducing AILI to optimize the therapeutic context. Mice in the AILI and HIRI models were euthanized at 10 and 6 h post‐induction, respectively. Serum and liver samples were collected for analysis.

### 5.4 Statistical Analysis

Data were expressed as mean ± SEM. Differences between two groups were assessed using a two‐tailed Student's t‐test. Statistical analyses were conducted using GraphPad Prism. A significance level of 0.05 was used for all statistical tests. Additional information on methods can be found in Supporting Information.

## Conflicts of Interest

The authors declare no conflicts of interest.

## Author Contributions

All authors contributed to the study's conception and design. Material preparation and data collection were performed by X.C., J.H. and J.D. Animal experiments were conducted with the assistance of Z.W., Z.W. and K. W. The clinical sample collection was completed by X.C., J.D., Z.S., X.Z., Z.G., X.M., S.W. and X.H. Data analysis was performed by X.C., J.H. and J.D. The first draft of the manuscript was written by X.C., J.H., and J.D., with reviews provided by Y.D., W.W., and M.Z. All authors read and approved the final manuscript.

## Supporting information




**Supporting File**: advs74943‐sup‐0001‐SuppMat.pdf.

## Data Availability

The data that support the findings of this study are available from the corresponding author upon reasonable request.
