# Supplementary material for: Liver‐Targeted Gallium‐Polyphenol Network by Disrupting the ROS/NETs/PANoptosis Axis for Precision Acute Liver Injury Therapy
Source: Adv Sci (Weinh). 2026 Mar 26;13(32):e24135. doi: 10.1002/advs.202524135 (PMC13252645; doi:10.1002/advs.202524135)
Supplement: Supplementary file 1 — Supporting File: advs74943‐sup‐0001‐SuppMat.pdf. [file ADVS-13-e24135-s001.pdf]

**Supplementary Information**

# Liver-targeted Gallium-Polyphenol Network by Disrupting the ROS/NETs/PANoptosis Axis for Precision Acute Liver Injury Therapy

Xiaopeng Cai[1,#], Jian He[2,3,#], Jingwen Deng[4,#], Ziwei Wang[3,5], Zhongshu Wu[3,5], Kaiyue Wang[6,7], Xiang Zheng[1], Zhenzhen Gao[1], Xi Ma[1], Shumei Wei[8], Xiangrong Hu[8], Yuan Ding[1,*], Weilin Wang[1,*], Min Zhou[2,3*]

*1 Hepatobiliary and Pancreatic Surgery, The Second Affiliated Hospital, Zhejiang University School of Medicine, Hangzhou, Zhejiang 310003, China.*

*2 Zhejiang University-University of Edinburgh Institute (ZJU-UoE Institute), and Liangzhu Laboratory, Zhejiang University School of Medicine, Zhejiang University, Haining, Zhejiang 314400, China.*

*3 Zhejiang University-Ordos City Etuoke Banner Joint Research Center, Zhejiang University, Haining, Zhejiang 314400, China*

*4 Department of Medical Oncology, Sir Run Run Shaw Hospital, School of Medicine, Zhejiang University, Hangzhou, Zhejiang 310016, China.*

*5 Zhejiang University-University of Edinburgh Institute (ZJU-UoE Institute), Zhejiang University, Haining 314400, China.*

*6 Department of Radiology, Sir Run Run Shaw Hospital of School of Medicine, Zhejiang University, Hangzhou, Zhejiang 310016, China.*

*7 Department of surgery and International Institutes of Medicine, The Fourth Affiliated Hospital, Zhejiang University School of Medicine, Yiwu, Zhejiang 322000, China*

*8 Departments of Clinical Pathology, The Second Affiliated Hospital of Medical College of Zhejiang University, Hangzhou, Zhejiang 310003, China.*

These authors contributed equally: Xiaopeng Cai, Jian He and Jingwen Deng.

Corresponding authors

E-mail: dingyuan@zju.edu.cn; wam@zju.edu.cn; zhoum@zju.edu.cn.

## Contents

**Supplementary Materials and Methods**

**Supplementary Figure 1.** Blocking ROS-mediated NETs protects against acute liver injury (ALI).

**Supplementary Figure 2.** NETs-mediated PANoptosis in acute liver injury (ALI).

**Supplementary Figure 3.** Synthesis and characteristics of Ga@Que.

**Supplementary Figure 4.** ROS scavenging assays and biosafety of Ga@Que.

**Supplementary Figure 5.** Protective effect of Ga@Que in acute liver injury (ALI).

**Supplementary Figure 6.** Ga@Que inhibits oxidative stress and reduces inflammation in hepatic ischemia-reperfusion injury (HIRI).

**Supplementary Figure 7.** Ga@Que blocks neutrophil migration and NETs formation in acute liver injury (ALI).

**Supplementary Figure 8.** Ga@Que reduces PANoptosis mediated by NETs in acute liver injury (ALI).

**Supplementary Table 1.** Comparison of Ga@Que with recent therapeutic strategies for acute liver injury (ALI)

**Supplementary Table 2.** Detailed information of antibodies

**Supplementary Table 3.** The detailed scoring rule for immunohistochemistry (IHC)

**Supplementary Table 4.** Primers used for RT-PCR

## Materials and methods

### Preparation and Characterization of Ga@Que

Ga@Que was prepared according to previous reports with modifications [1, 2]. Gallium nitrate ($Ga(NO_3)_3$, Makclin) aqueous solution and N-Methylpyrrolidone (Shanghai Aladdin) solution of polyphenol (Quercetin (Que), Gallic acid (GA), Tannic acid (TA), Pyrogallol (PG), Chrysin (Cry), Resveratrol (Rsv), Sigma-Aldrich and Shanghai Aladdin ) with the same concentration of 10 mg/mL were added to 10 mM phosphate buffer at a volume ratio of 2:1. The reaction was stirred for 30 minutes. The above mixture was dialyzed in ultrapure water and ultrafiltered to obtain gallium@polyphenol, which was stored at 4 °C for subsequent use. For rhodamine B (RhoB)-labelled Ga@Que, 1 mg of RhoB (Sigma-Aldrich) was added into the Que and Gallium mixture, and the other reaction steps were the same.

The hydrodynamic size and polydispersity index (PDI) of gallium@polyphenol in water or Ga@Que in the medium were obtained by a dynamic light scattering system (Malvern Panalytical Zetasizer Nano ZS90, UK). The transmission electron microscope captured the microscopic morphology and element distributions of Ga@Que (TEM, FEI Tecnai G2 F20, USA). The absorbance spectra of Ga@Que were recorded by a UV−vis spectrometer (SHIMADZU UV-2600, Japan). The crystal structure, elemental composition, and mass change data of Ga@Que were obtained by XRD diffractometer (Rigaku Smartlab), X-ray photoelectron spectroscopy (XPS, Thermo Scientific K-Alpha, USA), and thermogravimetric analyzer (TG, NETZSCH TG 209 F1 Libra), respectively. Ex vivo fluorescence imaging and quantitative analysis of the distribution of RhoB-labeled Ga@Que in the heart (H), liver (L), spleen (S), lung (Lu), and kidney (Ki) of mice after tail vein injection at different time points were measured by a Biospace Optima imaging system. Representative ROS (ABTS. +, OH, DPPH., and ONOO- ) scavenging assay was evaluated using the previously reported methods by UV−vis spectrometer [3-5].

### Histological and Immunohistochemical (IHC) Analysis

Liver tissues were fixed in 4%paraformaldehyde, embedded in paraffin, and

sectioned. Sections were stained with Hematoxylin and Eosin (H&E) for general histopathology. For IHC, antigen retrieval was performed, and sections were incubated overnight at 4°C with primary antibodies against MPO, PAD4, CitH3, NLRP3, ASC, Caspase-1, p-MLKL, Cleaved Caspase-3, Bax, and Bcl-2 (for details, see **Supplementary Table 2**). Immunoreactive scores (IRS) were calculated by multiplying the score for the percentage of positive cells by the score for staining intensity, as detailed in **Supplementary Table 3**.

**Biochemical Assays**

Serum was isolated by centrifuging blood samples at 8,000 rpm for 10 minutes. Levels of alanine aminotransferase (ALT), aspartate aminotransferase (AST), urea nitrogen (BUN), creatinine (Cr), creatine kinase (CK), and lactate dehydrogenase (LDH) were measured using commercial kits (Ningbo Purebio Biotechnology Co., Ltd.).

Liver homogenates were prepared and centrifuged at 3,000× g for 20 min. The supernatants were used to determine the activities of catalase (CAT), glutathione peroxidase (GPX), superoxide dismutase (SOD), and the level of malondialdehyde (MDA) using kits from Nanjing Jiancheng Bioengineering Institute. The concentrations of inflammatory cytokines (IL-1β, TNF-α, IL-10) in liver homogenates were quantified using ELISA kits (Solarbio Life Sciences).

**Western blotting (WB)**

Liver tissues were lysed in RIPA buffer (Beyotime) supplemented with PMSF protease inhibitor. Proteins were separated by SDS-PAGE and transferred onto PVDF membranes (Millipore). After blocking with 5% non-fat milk, membranes were incubated overnight at 4°C with primary antibodies (Supplementary Table 1), followed by incubation with appropriate HRP-conjugated secondary antibodies. For the expression of different proteins in the same blots, partly blotted membranes were incubated with Western blot fast stripping buffer (EpiZyme) followed by several washes, and treated as mentioned above. Protein bands were visualized using an Odyssey

imaging system (LI-COR).

### Electron Microscopy Examination

Mouse liver samples were dehydrated through a graded alcohol series, freeze-dried, and sputter-coated with gold for scanning electron microscopy (SEM, Hitachi Model SU-8010). For transmission electron microscopy (TEM), samples were prepared and observed using a Hitachi Model H-7650 TEM.

### RNA Extraction and Quantitative Real-Time PCR (qRT-PCR)

Total RNA was extracted from liver tissues using the RNA-Quick Purification Kit (Yeasen). cDNA was synthesized using HiScript II Reverse Transcriptase SuperMix (Vazyme). qRT-PCR was performed using ChamQ SYBR qPCR Master Mix (Vazyme) on a QuantStudio real-time PCR system. Gene expression was normalized to GAPDH, and relative fold changes were calculated using the $2^{(-\Delta\Delta Ct)}$ method. Primer sequences are listed in **Supplementary Table 4**.

### Bulk RNA-seq analysis

Total RNA was extracted from control, APAP, and APAP + Ga@Que groups using Trizol Reagent (Invitrogen Life Technologies). RNA concentration, quality, and integrity were assessed using a Bioanalyzer 2100 (Agilent) and NanoDrop spectrophotometer (ThermoFisher Scientific). Qualification criteria were: concentration > 50 ng/μL, RIN > 7.0, $OD_{260}/_{280}$ > 1.8. Strand-specific libraries were prepared using the Hieff NGS® Ultima Dual-mode RNA Library Prep Kit (Yeason). Library fragments were size-selected and quality-checked using the Agilent 2100 Bioanalyzer. Paired-end sequencing (150-200 bp reads) was performed by Astrocyte Technology Co. Ltd (Hangzhou, China) on an Illumina NovaSeq™ X Plus platform (PE150 strategy). Raw reads were processed with fastq. Clean reads were aligned to the reference genome using TopHat. Differentially expressed genes (DEGs) were identified ($P < 0.05$, $|\log_2(\text{fold change})| \geq 1$). DEG enrichment analysis was performed using clusterProfiler.

## Cell culture

Human hepatoma Hep G2 cells and human promyelocytic leukemia HL-60 cells were cultured in DMEM and RPMI-1640 medium, respectively, supplemented with 10% fetal bovine serum and 1% penicillin/streptomycin at 37°C in a 5% $CO_2$ incubator.

## Hep G2 cells live/dead, apoptosis and ROS detection

A tert-Butyl hydroperoxide (tBHP)-induced Hep G2 cell damage model was established as previously described [6]. Hep G2 cells were divided into five groups: Control, tBHP, tBHP + $Ga(NO_3)_3$ / Que, tBHP + NAC, and tBHP + Ga@Que. The last four groups were initially co-cultured with tBHP (50 μM) for 24 hours after cell adhesion, followed by a 24-hour co-incubation with $Ga(NO_3)_3$ / Que (25 μM), NAC (25 μM) and Ga@Que (25 μM), respectively. Finally, cells were stained with calcein-AM and PI for liver/dead detection (Yeasen BioTechnologies Co., Ltd.), FITC/PI double stain kit for apoptosis detection (Yeasen BioTechnologies Co., Ltd.) or DCFH-DA (Solarbio Life Science Co., Ltd.) for ROS detection.

## Co-culture Assay of dHL-60 cells with injured Hep G2 cells medium

*Differentiation of HL-60 cells into dHL-60 cells*: HL-60 cells were cultured in medium containing 1.25% dimethyl sulfoxide (DMSO) for 5 days to induce neutrophil-like differentiation.

*Induction of injured Hep G2 cells:* Using the method described above, we first established a tBHP-induced Hep G2 cell injury model, followed by a 24-hour co-culture with either Ga@Que (25 μM) or NAC (25 μM).

*Transwell migration of dHL-60 cells:* $1 \times 10^5$ dHL-60 cells in serum-free medium were seeded in the upper chamber of a transwell insert. Conditioned medium from injured Hep G2 cells was added to the lower chamber. After 72 hours, migrated cells on the lower membrane surface were fixed in 4% paraformaldehyde (PFA) for 20 min and stained with crystal violet for 5 min.

*Immunofluorescence of dHL-60 cells:* 1 × $10^5$ dHL-60 cells were seeded in a 6-well plate and cultured with conditioned medium from injured Hep G2 cells for 12 h. Cells were then washed (centrifugation), fixed with 4% PFA for 20 minutes, permeabilized with 0.1% Triton X-100 for 20 minutes, blocked with 10% fetal bovine serum for 30 minutes, and incubated overnight at 4°C with primary antibodies against CitH3 and MPO. Slides were stained with DAPI for 20 minutes. Representative images showing localization patterns are presented.

**Isolation of NETs and Hep G2 Stimulation**

The dHL-60 cells were stimulated with 100 nM PMA (Beyotime, Shanghai, China) for 4 hours to induce NETs formation. After incubation, the medium was carefully aspirated, and NETs adhering to the culture surface were gently washed with PBS. The resulting wash solution containing detached NETs was centrifuged at 400 × g for 10 minutes. The supernatant was collected, and NETs-associated DNA was quantified using the Quant-iT PicoGreen dsDNA reagent (Life Technologies) [7].

For cellular stimulation, Hep G2 cells were incubated with isolated NETs (100 ng/μl) for 24 hours. Where indicated, DNase I (100 U/mL) was added as an interventional treatment. Following stimulation, cells were subjected to immunofluorescence staining to evaluate the expression of key markers, including ASC (a pyroptosis marker), p-MLKL (a necroptosis marker), and Bax (an apoptosis marker).

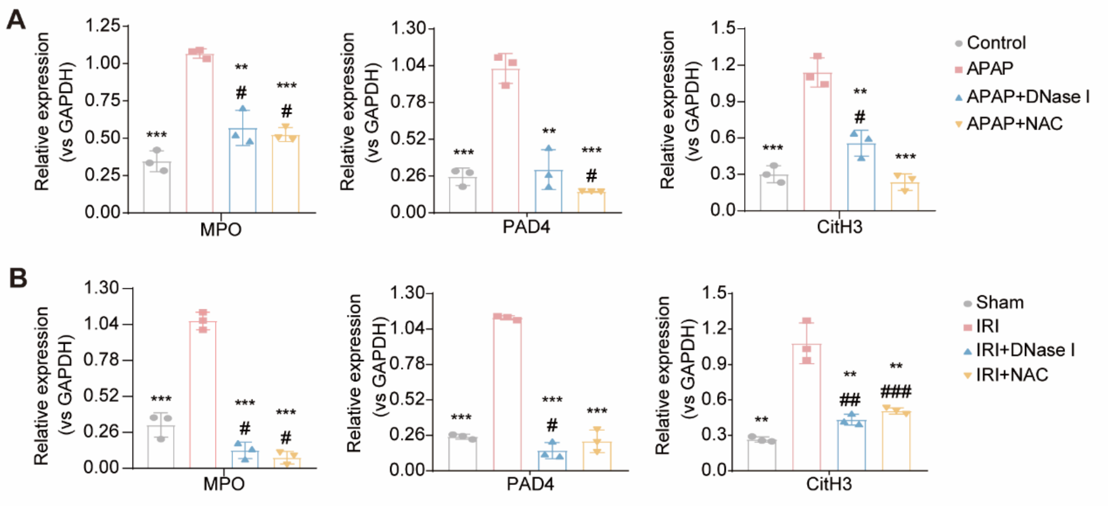


**Supplementary Figure 1. Blocking ROS-mediated NETs protects against acute liver injury (ALI). A-B.** Quantitative analysis of NETs markers (MPO, PAD4, CitH3) in liver tissues (n=3). *p < 0.05, **p < 0.01, ***p < 0.001 vs. APAP (or IRI) group; #p < 0.05, ##p < 0.01, ###p < 0.001 vs. Control (or Sham) group.

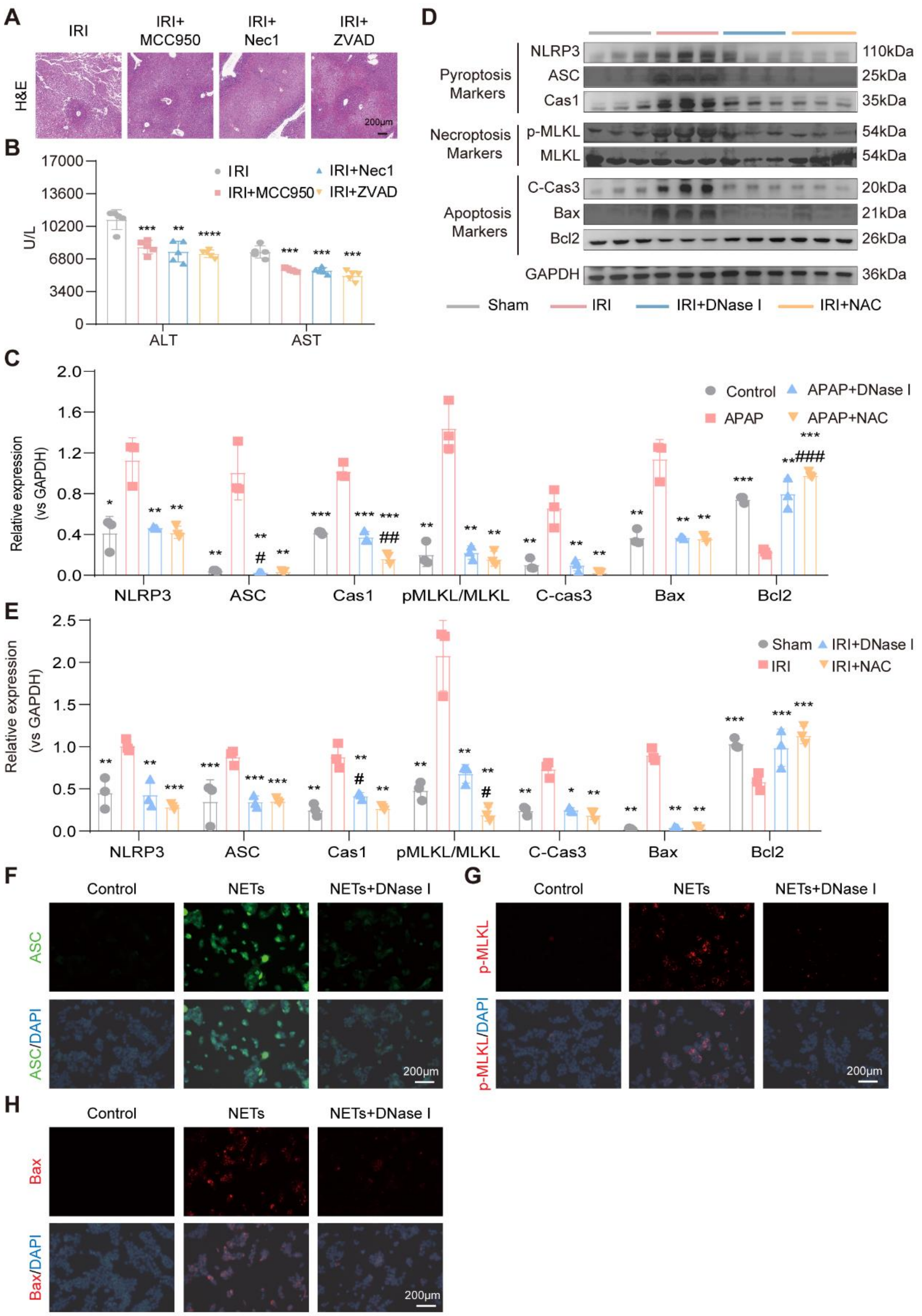


**Supplementary Figure 2. NETs-mediated PANoptosis in acute liver injury (ALI).** **A.** H&E staining after PANoptosis inhibitor administration in hepatic ischemia-reperfusion injury (HIRI) mice (n=5). **B.** Levels of alanine aminotransferase (ALT) and aspartate aminotransferase (AST) in HIRI mice, compared with the IRI model group

(n=5). **C.** Relative expression of PANoptosis biomarkers in acetaminophen-induced liver injury (AILI) mice (n=3). **D-E.** Expressions of PANoptosis biomarkers in HIRI mice (n=3). **F-H.** Representative images of immunoflurescence images of ASC (a pyroptosis marker), p-MLKL (a necroptosis marker), and Bax (an apoptosis marker). *p < 0.05, **p < 0.01, ***p < 0.001 vs. APAP (or IRI) group; #p < 0.05, ##p < 0.01, ###p < 0.001 vs. control (or Sham) group.

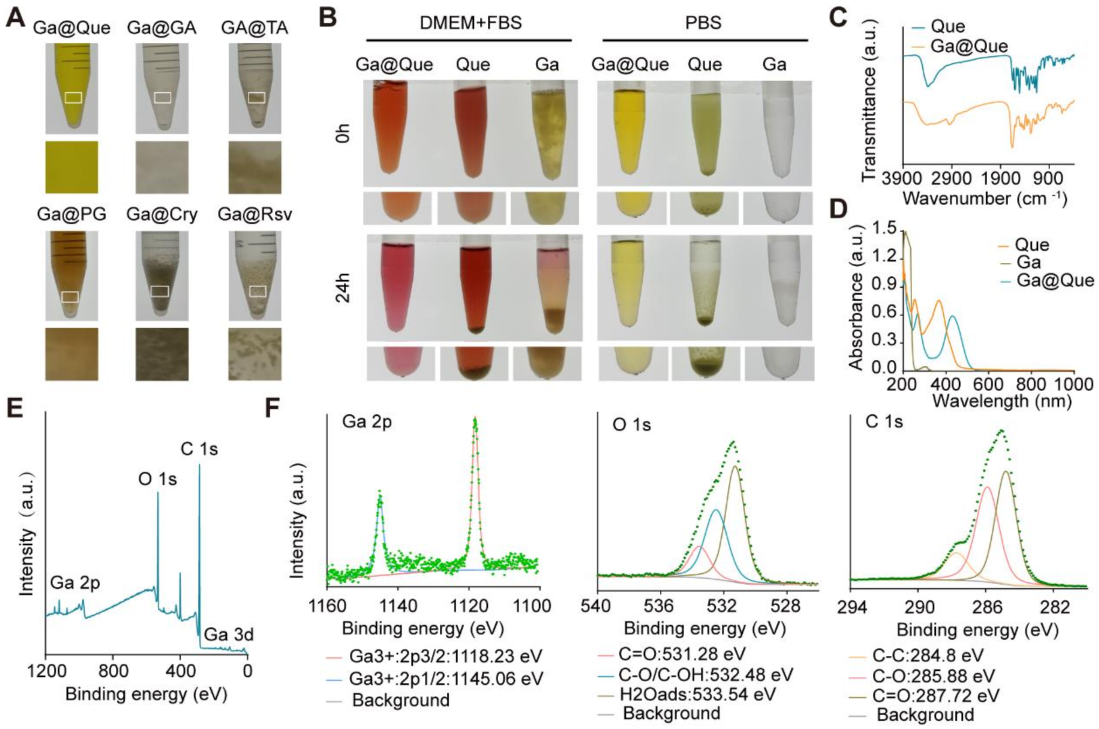


**Supplementary Figure 3. Synthesis and characteristics of Ga@Que. A.** Photographs of different polyphenol@gallium complexes. **B.** Photographs of Ga@Que in culture medium (DMEM+FBS) and PBS. **C.** FTIR spectra of Que and Ga@Que. **D.** UV-Vis-NIR absorbance spectra of Ga@Que components. **E-F.** XPS full spectrum and Ga 2p, O 1s, C 1s spectra of Ga@Que.

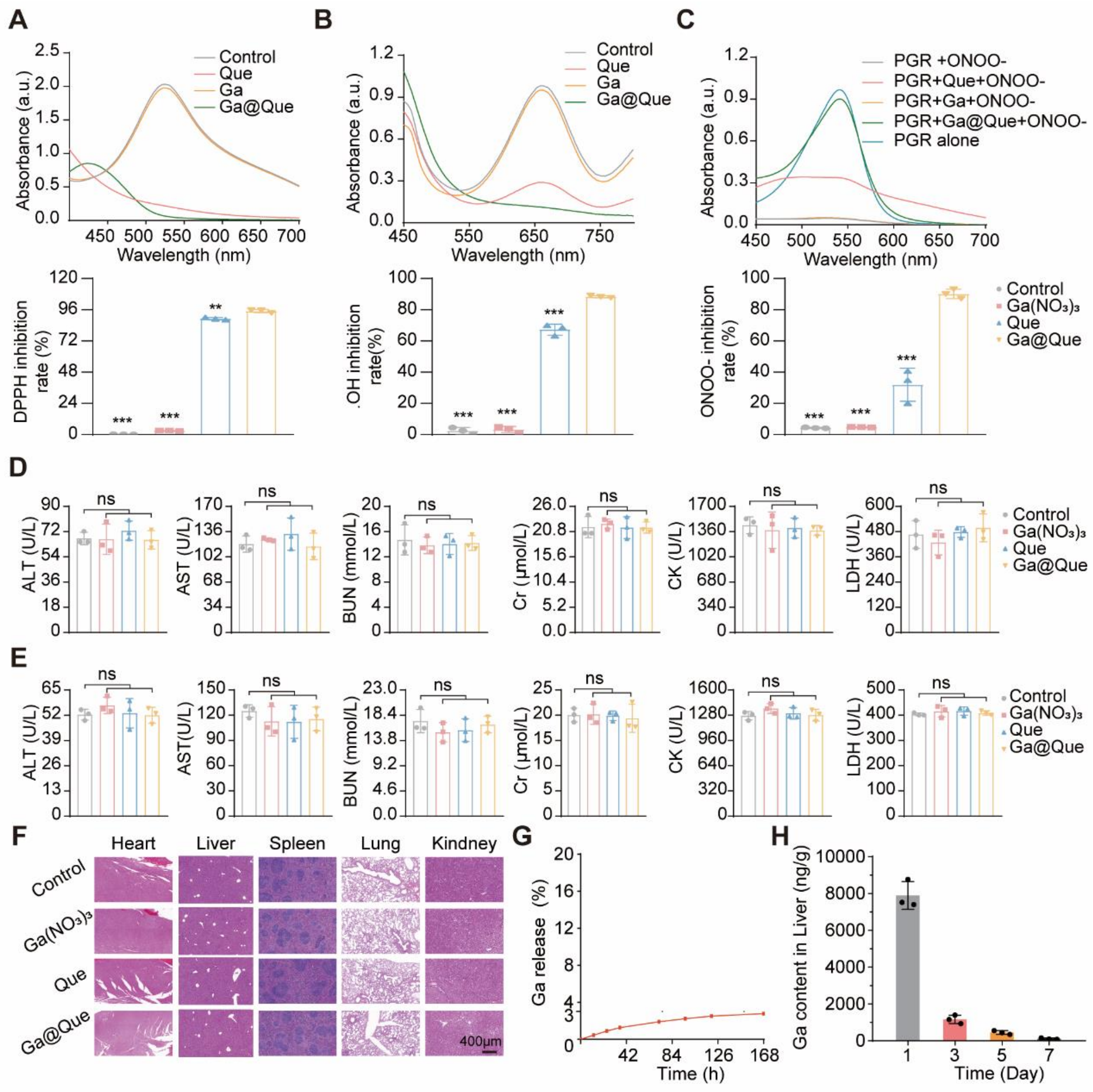


**Supplementary Figure 4. ROS scavenging assays and biosafety of Ga@Que. A-C.** Absorbance curves and corresponding scavenging rates of Ga@Que and its components against representative ROS: DPPH• (A), •OH (B), and $ONOO^-$ (C), compared with Ga@Que group (n=3). **D.** Short-term (24 h) biosafety assessment of $Ga(NO_3)_3$, Que, and Ga@Que by blood biochemistry (n=3). **E-F.** Long-term (1 week) biosafety assessment of $Ga(NO_3)_3$, Que, and Ga@Que by blood biochemistry and organ histopathology (n=3). **G.** The release behavior of Ga of Ga@Que in PBS (pH 7.4, 37°C) (n=3). **H.** The Ga content in the liver after intravenous injection of Ga@Que (n=3). **p< 0.01, ***p< 0.001.

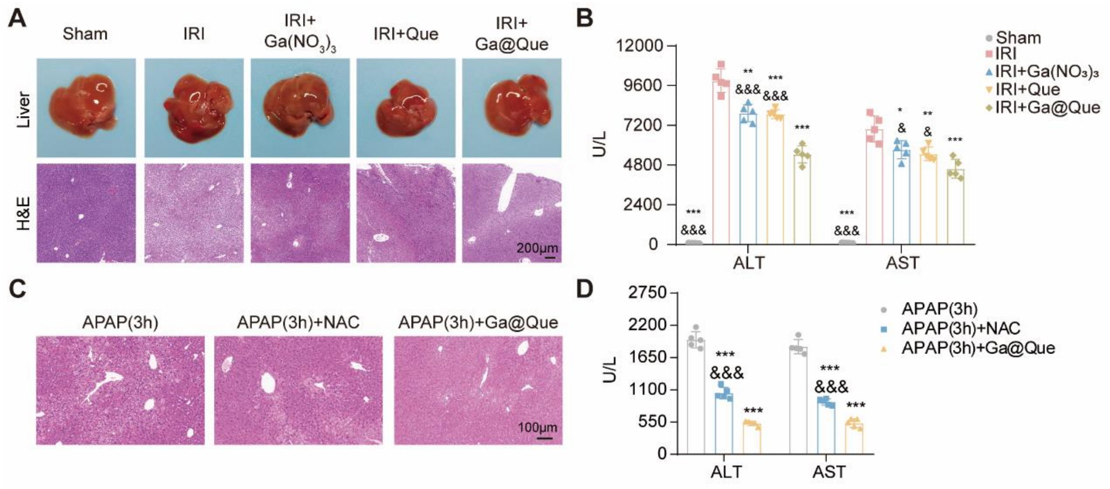


**Supplementary Figure 5. Protective effect of Ga@Que in acute liver injury (ALI). A.** Macroscopic appearance of the liver and H&E staining in HIRI mice (n=5). **B.** Alanine aminotransferase (ALT) and aspartate aminotransferase (AST) levels in HIRI mice (n=5). **C-D.** Liver H&E staining and ALT/AST levels were assessed at 3 hours after APAP administration, following treatment with NAC or Ga@Que, respectively. *p < 0.05, **p < 0.01, ***p < 0.001 vs. IRI group; &p < 0.05, &&&p < 0.001 vs. IRI + Ga@Que group (or APAP(3h)+Ga@Que).

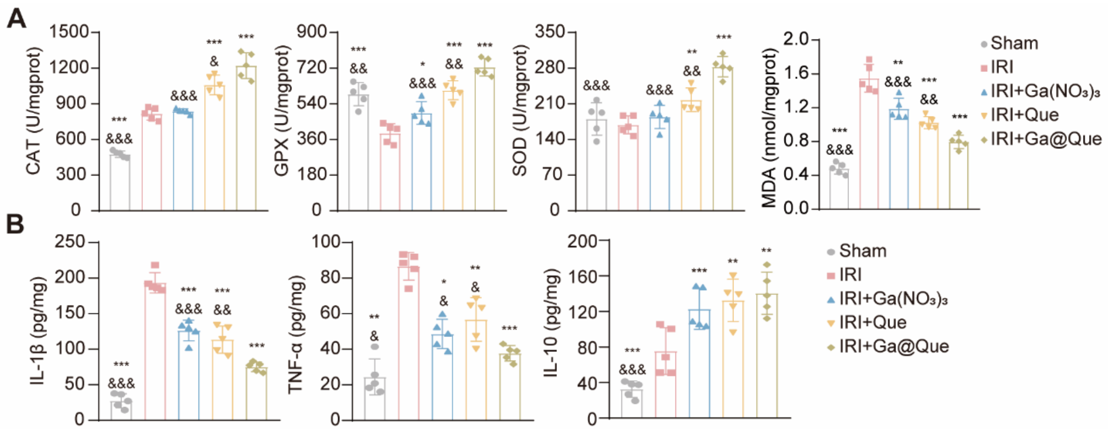


**Supplementary Figure 6. Ga@Que inhibits oxidative stress and reduces inflammation in hepatic ischemia-reperfusion injury (HIRI). A.** Levels of oxidases (CAT, GPX, SOD) and the oxygen-free radical metabolite MDA in liver tissue of HIRI mice (n=5). **B.** Ga@Que reduces inflammatory cytokine levels in HIRI mice (n=5). *p < 0.05, **p < 0.01, ***p < 0.001 vs. IRI group; &p < 0.05, &&p < 0.01, &&&p < 0.001 vs. IRI + Ga@Que group.

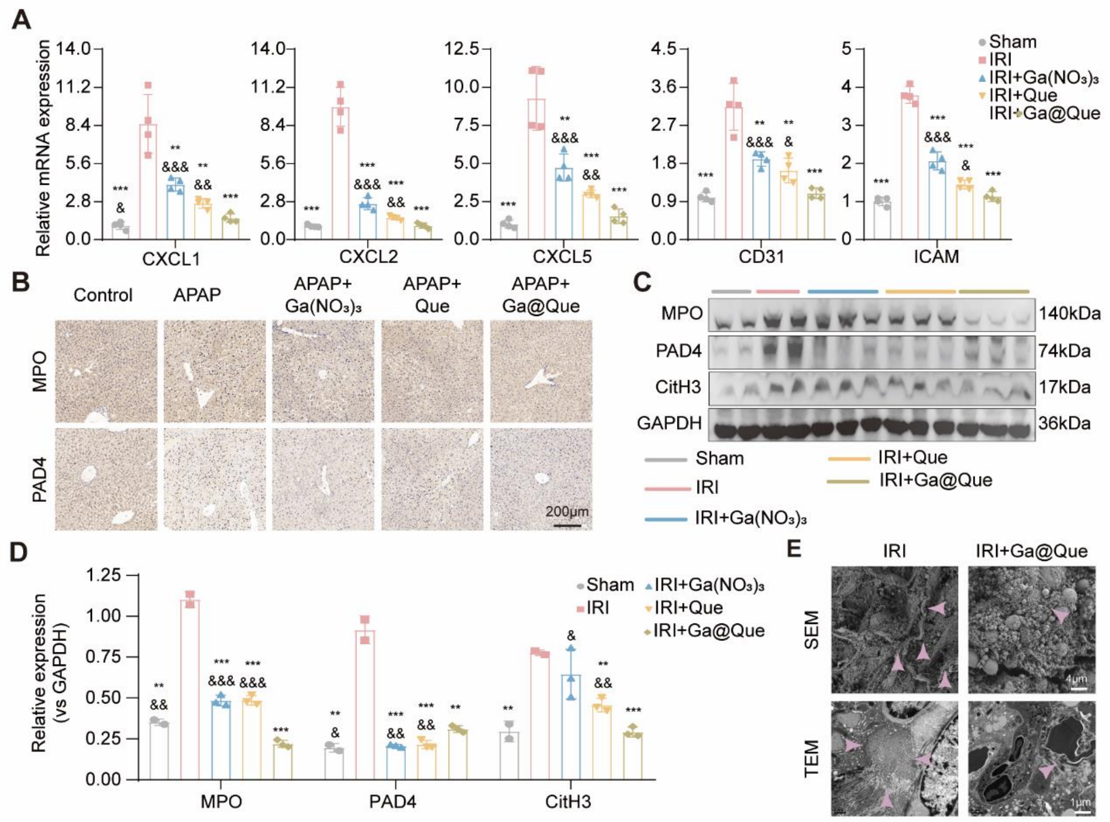


**Supplementary Figure 7. Ga@Que blocks neutrophil migration and NETs formation in acute liver injury (ALI). A.** Relative mRNA expression of neutrophil chemokines (CXCL1, CXCL2, CXCL5) and integrins (CD31, ICAM) in liver tissue of hepatic ischemia-reperfusion injury (HIRI) mice (n=4). **B.** Immunohistochemical staining of MPO and PAD4 in acetaminophen-induced liver injury (AILI) mice. **C-D.** Expressions of MPO, PAD4, and CitH3 in HIRI mice after Ga@Que administration (n=3). **E.** Observation of NETs using transmission electron microscopy (TEM) and scanning electron microscopy (SEM). $^{**}p < 0.01$, $^{***}p < 0.001$ vs. IRI group; &$p < 0.05$, &&$p < 0.01$, &&&$p < 0.001$ vs. IRI + Ga@Que group.

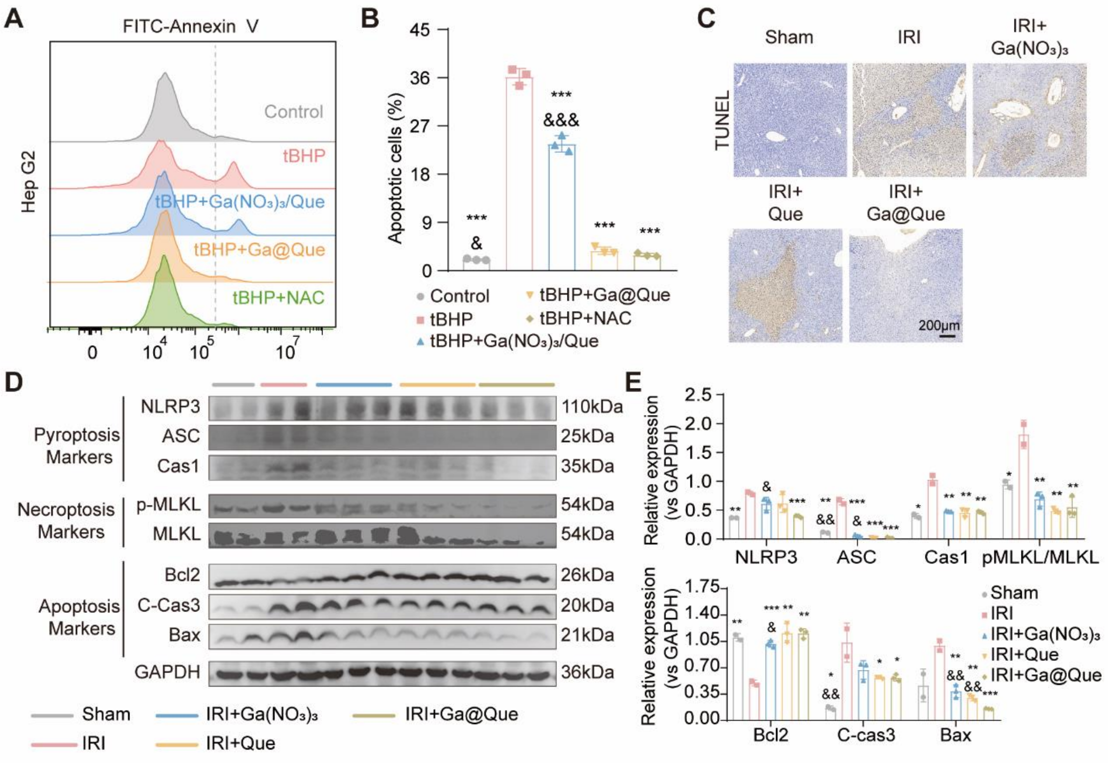


**Supplementary Figure 8. Ga@Que reduces NETs-mediated PANoptosis in acute liver injury (ALI). A-B.** Ga@Que reduces apoptosis in Hep G2 cells (n=3). **C.** Immunohistochemical staining of PANoptosis biomarkers in hepatic ischemia-reperfusion injury (HIRI) mice. **D-E.** Expressions of PANoptosis biomarkers in liver tissue of HIRI mice after Ga@Que administration (n=3). *p < 0.05, **p < 0.01, ***p < 0.001 vs. tBHP (or IRI) group; &p < 0.05, &&p < 0.01, &&&p < 0.001 vs. tBHP (or IRI) + Ga@Que group.

**Supplementary Table 1. Comparison of Ga@Que with recent therapeutic strategies for acute liver injury (ALI)**

| Reference | Therapeutic Approach | Mechanism | Liver Injury Model | Key Findings | Advantages of Our Study |
|---|---|---|---|---|---|
| This study | Liver-targeted Ga@Que via coordination-driven self-assembly | Scavenges ROS, suppresses neutrophil infiltration and NETs formation, attenuates PANoptosis; synergistic effects from Ga and quercetin | AILI and HIRI in mice; validated with multi-omics and clinical samples | Significant liver accumulation; reduced ALT/AST, inflammation, oxidative stress; outperformed individual components; biosafety confirmed | N/A (our study) |
| [8] | Natural polyphenol (catechin) | Inhibits ferroptosis via xCT/GPX4 pathway; reduces ROS/MDA, GSH depletion; inhibits STAT1 to attenuate inflammation | APAP-induced ALI in mice and HepG2 cells | Attenuated liver injury, oxidative stress, and inflammation; identified catechin as a ferroptosis inhibitor | Ga@Que addresses a broader pathogenic axis (ROS/NETs/PANoptosis) beyond ferroptosis; uses nanocomposite for improved bioavailability and liver targeting, showing efficacy in multiple ALI etiologies |
| [9] | Redox-responsive nanoparticles loaded with cannabidiol (CBD NPs) based on pullulan-deoxycholic acid-α-lipoic acid polymer | Redox-responsive release; reduces oxidative stress, inflammation; liver targeting via polymer | $CCl_4$-induced ALI in mice; $H_2O_2$-induced damage in HepG2 cells | Reduced ALT/AST, MDA, inflammatory cytokines; protected histology; strong liver accumulation | Our approach integrates metal-phenolic networks for synergistic ROS scavenging and immunomodulation; targets novel axis (NETs/PANoptosis); validated in clinical samples and two models |
| [10] | HMGB1 gene-silenced large peritoneal macrophages (GLPMs) using siRNA in C12-200 lipid nanoparticles | Selective tropism of GLPMs to injured liver; silences HMGB1 to prevent pro-inflammatory shift and mitigate inflammation | APAP-induced ALI in mice; tracked via diffuse in vivo flow cytometry (DiFC) | Reduced liver injury, cytokines; protected from APAP damage; no effect with hepatocyte-targeted siRNA | Our Ga@Que is a non-cellular, "off-the-shelf" nanotherapeutic with easier scalability; directly targets ROS/NETs/PANoptosis upstream; broader multi-omics validation |
| [11] | Allogeneic alternatively-activated macrophages (AAMs) | Promote resolution of necrosis and hepatocyte proliferation; phase 1 trial protocol for safety/tolerability | Paracetamol-induced acute liver injury in humans (phase 1 randomized trial) | Protocol for dose-escalation; aims to evaluate safety, biomarkers, and activity | Our preclinical Ga@Que provides mechanistic insights (ROS/NETs/PANoptosis) for potential future trials; non-cellular, targeted nano-delivery reduces immunogenicity risks |
| [12] | Dual-specific CRISPR-Cas nanosystem (Cas9 or CasRx) with macrophage membrane coating and liver-specific promoter | Liver-targeted DNA/RNA editing; disrupts inflammatory genes; macrophage membrane for inflammation homing | Liver fibrosis and acute injury models in mice (e.g., $CCl_4$, APAP) | Specific editing in liver; reduced inflammation/fibrosis; minimal off-target effects | Our Ga@Que is simpler (no gene editing risks like genotoxicity); targets broad axis via small-molecule nanocomposite; easier translation without viral/non-viral editing concerns |

**Supplementary Table 2.** Detailed information of antibodies

| Antibody | Host | Reactivity | Category number | Brand |
|---|---|---|---|---|
| Anti-MPO | Rabbit | Human, mouse | 22225-1-AP | proteintech |
| Anti-PAD4 | Rabbit | Human, mouse | ab96758 | Abcam |
| Anti-CitH3 | Mouse | Human, mouse | 13754-1-AP | proteintech |
| Anti-NLRP3 | Mouse | Human, mouse | 68102-1-Ig | proteintech |
| Anti-ASC | Rabbit | Human, mouse | 10500-1-AP | proteintech |
| Anti-Caspase1 | Rabbit | Human, mouse | 22915-1-AP | proteintech |
| Anti-Phospho-MLKL | Rabbit | Human, mouse | AF7420-100 | Affinity Biosciences |
| Anti-MLKL | Mouse | Human, mouse | 66675-1-Ig | proteintech |
| Anti-Bcl2 | Rabbit | Human, mouse | 26593-1-AP | proteintech |
| Anti-Cleaved Caspase 3 | Rabbit | Human, mouse | 25128-1-AP | proteintech |
| Anti-Bax | Rabbit | Human, mouse | 50599-2-Ig | proteintech |
| Anti-Vinculin | Rabbit | Human, mouse | ab129002 | Abcam |
| Anti-GAPDH | Rabbit | Human, mouse | 10494-1-AP | proteintech |

**Supplementary Table 3.** The detailed scoring rule for immunohistochemistry (IHC)

| Percentage of positive cells (a) | | Staining intensity grading (b) | |
|---|---|---|---|
| Percentage (%) | Score | Intensity | Score |
| ﹤5% | 0 | None | 0 |
| 5-25% | 1 | Light brown yellow | 1 |
| 25-50% | 2 | Brown yellow | 2 |
| 50-75% | 3 | Dark brown yellow | 3 |
| 75-100% | 4 | | |

Histoscore=a*b

**Supplementary Table 4.** Primers used for RT-PCR

| Genes | Forward primer sequence (5’-3’) | Reverse primer sequence (5’-3’) |
|---|---|---|
| CXCL1 | GACCATGGCTGGGATTCACC | GACTTCGGTTTGGGTGCAGT |
| CXCL2 | CATAGCCACTCTCAAGGGCG | GATGATTTTCTGAACCAGGGGG |
| CXCL5 | CCCTTCCTCAGTCATAGCCG | CTATGACTTCCACCGTAGGGC |
| CD31 | AGGTGAAGGTGCATGAGTCC | CTAATGTGCAGCTGGTCCCC |
| ICAM | CTGGGCTTGGAGACTCAGTG | CCACACTCTCCGGAAACGAA |
| GAPDH | GTGTTCCTACCCCCAATGTG | ATTGTCATACCAGGAAATGAGC |

**References:**

[1] Xu W, Lin Z, Pan S, Chen J, Wang T, Cortez-Jugo C, Caruso F. Direct Assembly of Metal-Phenolic Network Nanoparticles for Biomedical Applications[J]. Angewandte Chemie International Edition. 2023,62(45).

[2] Wang Q, He J, Qi Y, Ye Y, Ye J, Zhou M. Ultrasound-enhanced nano catalyst with ferroptosis-apoptosis combined anticancer strategy for metastatic uveal melanoma[J]. Biomaterials. 2024,305:122458.

[3] Wang C, Zhao M, Xie J, Wang H, Gu Z, Sun F. Colon-Targeted Release of Gel Microspheres Loaded with Antioxidative Fullerenol for Relieving Radiation-Induced Colon Injury and Regulating Intestinal Flora[J]. Advanced Healthcare Materials. 2023,12(30).

[4] Yin W, Yu J, Lv F, Yan L, Zheng LR, Gu Z, Zhao Y. Functionalized Nano-MoS2 with Peroxidase Catalytic and Near-Infrared Photothermal Activities for Safe and Synergetic Wound Antibacterial Applications[J]. ACS Nano. 2016,10(12):11000-11.

[5] Xie J, Wang N, Dong X, Wang C, Du Z, Mei L, Yong Y, Huang C, Li Y, Gu Z, Zhao Y. Graphdiyne Nanoparticles with High Free Radical Scavenging Activity for Radiation Protection[J]. ACS Applied Materials & Interfaces. 2018,11(3):2579-90.

[6] Zheng Z, Xie J, Ma L, Hao Z, Zhang W, Li L. Vitamin D Receptor Activation Targets ROS-Mediated Crosstalk Between Autophagy and Apoptosis in Hepatocytes in Cholestasic Mice[J]. Cell Mol Gastroenterol Hepatol. 2023,15(4):887-901.

[7] Zeng FL, Zhang Y, Wang ZH, Zhang H, Meng XT, Wu YQ, Qian ZZ, Ding YH, Li J, Ma TT, Huang C. Neutrophil extracellular traps promote acetaminophen-induced acute liver injury in mice via AIM2[J]. Acta Pharmacol Sin. 2024 Apr 8.

[8] Su Y, Zeng Y, Zhou M, Liao M, Qin P, Wu R, Han J, Liang X, Wang Z, Jiang J, Yu Z, Huang X, Ding K, Guo P, He Y, Du Y, Duan T, Yuan H, Ge Y, Chen A, Xiao W. Natural Polyphenol-Mediated Inhibition of Ferroptosis Alleviates Oxidative Damage and Inflammation in Acute Liver Injury[J]. Biomater Res. 2025,29:0167.

[9] Zhang X, Yi X, Gao X, Li Y, Shen X. Liver-Targeted Nanoparticles Loaded with

Cannabidiol Based on Redox Response for Effective Alleviation of Acute Liver Injury[J]. Foods. 2024 Aug 4,13(15).
[10] Oza D, Ivich F, Deprey K, Bittner K, Bailey K, Goldman S, Yu M, Niedre M, Tu HC, Amiji MM. Treatment of Acute Liver Injury through Selective Tropism of High Mobility Group Box 1 Gene-Silenced Large Peritoneal Macrophages[J]. ACS Nano. 2025 Apr 1,19(12):12102-18.
[11] Humphries C, Addison M, Aithal G, Boyd J, Briody L, Campbell JDM, Candela ME, Clarke E, Coulson J, Downing-James N, Fontana RJ, Geddes A, Grahamslaw J, Grant A, Heye A, Hutchinson JA, Jones A, Mitchell F, Moore J, Riddell A, Rodriguez A, Thomas A, Tucker G, Walker K, Weir CJ, Woods R, Zahra S, Forbes SJ, Dear JW. Macrophage Therapy for Acute Liver Injury (MAIL): a study protocol for a phase 1 randomised, open-label, dose-escalation study to evaluate safety, tolerability and activity of allogeneic alternatively activated macrophages in patients with paracetamol-induced acute liver injury in the UK[J]. BMJ Open. 2024 Dec 9,14(12):e089417.
[12] Xu X, Tang H, Guo J, Xin H, Ping Y. A dual-specific CRISPR-Cas nanosystem for precision therapeutic editing of liver disorders[J]. Signal Transduct Target Ther. 2022 Aug 12,7(1):269.
